# Integrating bioprinting, cell therapies and drug delivery towards in vivo regeneration of cartilage, bone and osteochondral tissue

**DOI:** 10.1007/s13346-023-01437-1

**Published:** 2023-10-26

**Authors:** Anna Abbadessa, Alfredo Ronca, Aurelio Salerno

**Affiliations:** 1 Center for Research in Molecular Medicine and Chronic Diseases (CiMUS), IDIS Research Institute, Universidade de Santiago de Compostela, 15782 Santiago de Compostela, Spain; 2 Department of Pharmacology, Pharmacy and Pharmaceutical Technology, School of Pharmacy, Universidade de Santiago de Compostela, Campus Vida, Santiago de Compostela, Spain; 3 Institute of Polymers, Composites and Biomaterials, National Research Council, 80125 Naples, Italy; 4 Department of Chemical, Materials and Production Engineering, University of Naples Federico II, 80125 Naples, Italy

**Keywords:** Bioprinted scaffolds, Bone, Cartilage, Osteochondral tissue, Growth factors, Spatio-temporal drug release

## Abstract

The biological and biomechanical functions of cartilage, bone and osteochondral tissue are naturally orchestrated by a complex crosstalk between zonally dependent cells and extracellular matrix components. In fact, this crosstalk involves biomechanical signals and the release of biochemical cues that direct cell fate and regulate tissue morphogenesis and remodelling in vivo. Three-dimensional bioprinting introduced a paradigm shift in tissue engineering and regenerative medicine, since it allows to mimic native tissue anisotropy introducing compositional and architectural gradients. Moreover, the growing synergy between bioprinting and drug delivery may enable to replicate cell/extracellular matrix reciprocity and dynamics by the careful control of the spatial and temporal patterning of bioactive cues. Although significant advances have been made in this direction, unmet challenges and open research questions persist. These include, among others, the optimization of scaffold zonality and architectural features; the preservation of the bioactivity of loaded active molecules, as well as their spatio-temporal release; the in vitro scaffold maturation prior to implantation; the pros and cons of each animal model and the graft-defect mismatch; and the in vivo non-invasive monitoring of new tissue formation. This work critically reviews these aspects and reveals the state of the art of using three-dimensional bioprinting, and its synergy with drug delivery technologies, to pattern the distribution of cells and/or active molecules in cartilage, bone and osteochondral engineered tissues. Most notably, this work focuses on approaches, technologies and biomaterials that are currently under in vivo investigations, as these give important insights on scaffold performance at the implantation site and its interaction/integration with surrounding tissues.

**Graphical Abstract:**

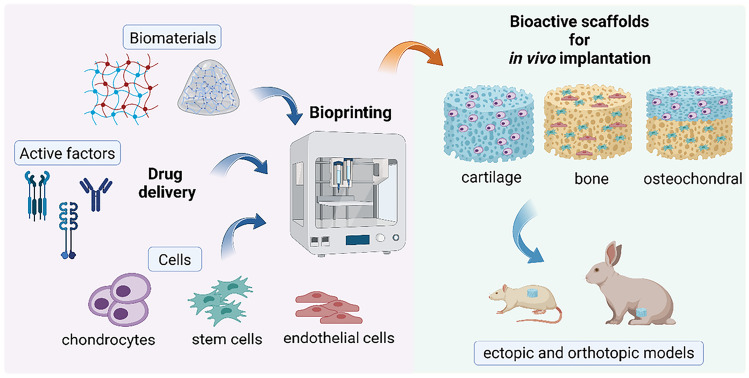

## Introduction

Three-dimensional (3D) bioprinting technologies have revolutionized the field of tissue engineering (TE) and biomedicine as they allow to build customized, patient-specific multifunctional bioscaffolds to repair damaged tissues and organs [1–4]. To achieve this aim, bioprinting techniques use virtual computer-aided design (CAD) models obtained from medical imaging and translate these models into 3D biomedical devices [1–4]. These technologies were developed and implemented in the biomedical field at the beginning of the 2000s and were defined as the “techniques using material transfer processes to design and assemble living cells, biomolecules, and biodegradable biomaterials according to a specific 3D configuration to perform one or more biological functions” [1, 2]. Due to the evolution of materials science and processing technologies, to date, bioprinting also includes photopolymerization processes that do not require material transfer [5, 6].

Bioprinted scaffolds can be designed and engineered towards the regeneration of a large variety of soft and hard tissues with clinically relevant size and geometrical features [7, 8]. In this context, 3D bioprinting has recently opened new avenues for upscaling the fabrication of bioscaffolds towards achieving the biological and biomechanical requirements of musculoskeletal tissues [7]. The musculoskeletal system is essential for protecting organs, enabling locomotion and regulating numerous cellular and metabolic functions [9]. However, in vivo regeneration of musculoskeletal tissues has not yet been achieved using scaffold-mediated TE approaches, given that the tissues of the musculoskeletal system are characterized by a hierarchical complex interplay between cells and extracellular matrix (ECM) [10].

In vitro evaluation of scaffolds is subjected to several limitations arising from the reduced complexity of the in vitro culture models, the absence of immune or inflammatory response as well as the impossibility to reproduce the complex cascade of events occurring after in vivo implantation [11, 12]. These include the interaction with body fluids (e.g. blood and synovial fluid) and recruitment of multiple cells that participate in the wound healing [11]. In contrast, in vivo animal studies of bioprinted scaffolds allow the assessment of biomaterials under different loading conditions and for extended time durations and, therefore, are the necessary step to assess the suitability of scaffolds for clinical translation.

This review describes recent advances in the 3D bioprinting of bone, cartilage and osteochondral (OC) tissue, which form an essential part of the musculoskeletal system. In particular, the attention is focused on scaffold-based approaches that integrated biomaterials with cells and/or active molecules and that were supported by in vivo validation of the bioconstructs. The strategy of engineering cell-laden scaffolds is applied to endow the scaffolds with continuous physiological functions, as cells constantly sense the environment and dynamically transmit signals, which is crucial for the synthesis of new ECM and, hence, for tissue regeneration [13]. In addition to or in lieu of encapsulating autologous cells into bioprinted constructs, the use of bioactive molecules, such as growth factors, may enable or intensify patient’s native cell recruitment, proliferation and biosynthesis [14]. The incorporation of cells and/or growth factors in bioprinted constructs, better if performed in a spatially controlled fashion, is therefore a prerequisite to accelerate cellular activities necessary for tissue regeneration in vivo. Here, one of the major challenges is to preserve the long-term viability and durable physiological functions of cells and biomolecules in the scaffolds during manufacturing and over the entire new tissue regeneration time scale [2].

In this rapidly evolving area, a promising new trend relies on the integration of drug delivery systems into 3D bioprinted scaffolds [2, 15]. As discussed in this review, this approach holds great potential; however, to successfully merge these two different technologies, several critical aspects must be carefully considered. Firstly, the drug dosing needs thorough revision, as the 3D printing process itself can impact the drug entrapment and stability within the scaffold. For example, the generation of shear forces during 3D printing could affect the overall integrity of the drug delivery system and the stability of the entrapped drug [16]. Understanding the impact of these mechanical forces is essential for fine-tuning printing parameters, selecting the most appropriate bioink and ensuring drug stability. Moreover, post-printing procedures, such as washing steps, may trigger premature release of the entrapped drug. This aspect must be addressed by accurately optimizing post-printing protocols. Furthermore, the 3D bioprinted scaffold itself may act as an additional barrier to the free diffusion of the released drug. Hence, studying the release kinetics of the entrapped drug in the presence of the scaffold is crucial. Additionally, it is crucial to study how the inclusion of drug delivery systems into 3D bioprinted scaffolds affects scaffold mechanical properties and long-term stability [2]. Finally, when cells are also included, their interaction with the delivery system must be studied to exclude undesired effects, such as the cellular uptake of nano-sized particles. The accurate knowledge of all these aspects is paving the way for new developments in the field.

Several articles have been published in the recent years about bioprinting for scaffold fabrication and described in details different aspects related to the development and properties of bioinks [17–19], the advantages and drawbacks of bioprinting techniques [20–22], the spatio-temporal control of growth factors by 3D printing [2, 16] and the application of bioprinted scaffolds in tissues like bone, cartilage and OC tissue [23–26]. However, to the best of our knowledge, this is the first review article that integrated and critically reviewed recent advances on bioprinting scaffolds bioactivated with cells and/or biomolecules towards in vivo regeneration of bone, cartilage and OC tissue. More in detail, in this review, we included papers of the last 5 years that describe in vivo evaluation of 3D printed *bioinks*, i.e. biomaterials containing living cells and *biomaterial inks* (i.e. “aqueous formulations of polymers or hydrogel precursors that contain biological factors”, according to the definitions proposed by Groll et al. [27]). In line, we excluded papers that describe scaffolds, where the addition of cells and/or active factors occurs after the 3D printing process. In the first part of the review, we summarize the 3D bioprinting techniques used to fabricate scaffolds that are currently under in vivo evaluation. In the second part of the review, we report and critically discuss the advances in cartilage, bone and OC tissue. Finally, in the last part, we analyse the challenges and open questions which are crucial to bear in mind for a realistic transition to the clinical setting.

## Anatomy and physiology of cartilage, bone and OC tissue

### Cartilage

Articular cartilage (AC) is an anisotropic tissue whose function is to lower articulation friction, withstand high cyclic loads without degenerative changes and allow load transmission through the surrounding bone. The composition and structure of AC varies with depth [28–30]. Overall, AC is a hypocellular tissue with almost 2% of its volume made of chondrocytes (CCs), while the main tissue components are water (65 to 85%), collagen (CoL) type II and proteoglycans [28]. The spatial distribution of these components varies with depth, and it is possible to identify three main zones: the superficial, the middle and the deep zones. The superficial zone is composed of an acellular sheet of CoL on top of a thicker layer of tightly packed, flattened CCs oriented parallelly to the surface. The distribution and orientation of CoL fibres act in synergy to proteoglycans, such as lubricin, and synovial fluid constituents in reducing AC friction, finally increasing cartilage surface smoothness and shear resistance [28]. Within the intermediate zone, CCs are more spherical and have an active role in ECM biosynthesis. The CoL fibrils are obliquely oriented with respect to the articular surface while there is more proteoglycan amount and less CoL and water content. In the deeper zone, CCs are rounded and stacked in columns perpendicular to the articular surface, following the arrangement of CoL fibrils, and here, cells have the highest ECM synthesis activity [29].

### Bone

Bone is a dynamic tissue that provides structural support to the body, enables movement and locomotion, safeguards important internal organs and structures, maintains mineral homeostasis and acid–base balance, acts as a reservoir for growth factors and cytokines and creates the conditions for haematopoiesis in the marrow spaces [31, 32]. Bone is a hierarchical composite material consisting of a mineral phase, namely hydroxyapatite (Hap) (Ca_10_(PO_4_)_6_(OH)_2_), and an organic phase composed of CoL type I (∼ 90%), non-collagenous proteins (∼ 5%), lipids (∼ 2%) and water [33–36]. Importantly, the relative amount of each of these constituents varies with age, site, gender, ethnicity and health status [33].

**Fig. 1:**
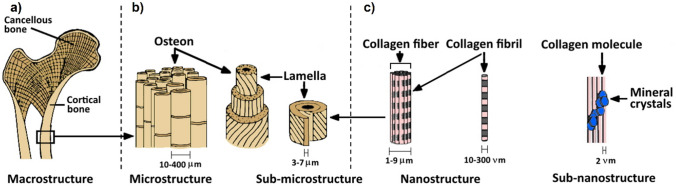
Schematic illustration of the hierarchical structure of bone. **a** At the macrostructural level, bone is composed of cortical bone and cancellous bone. **b** At the microstructural level, the cortical bone is made up of repeated units of osteon, which is characterized by 20–30 concentric layers of CoL fibres, called lamellae. The lamellae surround the central canal and contain various blood vessels and nerves. **c** At the nanostructural level, there is a large number of CoL fibres, which are composed of periodic CoL fibrils and gaps between the CoL molecules. The calcium phosphate crystals and non-collagenous organic proteins are embedded in these gaps between CoL molecules [38]

From an anatomical point of view, we have long bones, short bones, flat bones, sesamoid bones and irregular bones, whereas from a structural point of view, bone can be divided into cortical and cancellous bones [37]. Cortical bone is constituted by close packets of osteons, cylindrical (Haversian) systems with a central channel composed of a blood vessel and surrounded by concentric rings (lamellae) of bone matrix (Fig. 1). In contrast, cancellous bone is less dense and is structured in plates (trabeculae) offering a larger surface area-to-mass ratio, making it an effective structure for homeostasis and haematopoiesis as well as imparting flexibility in load-bearing bones.

### OC tissue

The OC tissue is the interfacial structure between bone and hyaline cartilage of an articular joint, and it is characterized by the gradual transition from the superficial cartilage to the inner subchondral bone region. The transition between AC and bone tissue is mediated by the calcified cartilage zone, characterized by a decrease in the amount of CoL fibres, and the presence of a significant amount (more than 60% in dry weight) of calcium phosphate in the form of Hap. This layer facilitates the stress distribution and reduces possible delamination owing to horizontal shear stresses [29, 30]. The AC is anchored to the subchondral bone plate by CoL fibrils that penetrate the calcified cartilage zone. The vertically orientated CoL fibrils extend from the deep zone of cartilage to the calcified cartilage through a wavy tidemark, but do not enter in contact with the subchondral bone [39]. The subchondral bone is immediately distal to the mineralized cartilage zone and lies above the subchondral trabecular or cancellous bone. The role of subchondral bone is double. Since cartilage is largely avascular, the vascularized subchondral bone provides nutrients and oxygen to sustain articular CCs. Furthermore, subchondral bone ensures stabilization and load support for the knee joint where it distributes axial loads transmitted through the cartilage and meniscus, finally preserving these cartilaginous structures [30, 39].

## Bioprinting techniques for in vivo bone, cartilage and OC tissue regeneration

One of the advantages of using 3D bioprinting is the ability to precisely control the positioning of living cells, bioactive molecules and ECM components, to mimic the hierarchal organization of bone, cartilage and OC tissue [40]. This approach allows the recapitulation of naturally occurring morphological characteristics, as well as biochemical composition, stiffness and overall organizational complexity [22]. To this aim, 3D printing is being utilized for the development of multimaterial scaffolds with a gradient distribution of biomaterials, cells and active factors, as extensively discussed in sections ‘In vivo advances in the 3D bioprinting of cartilage’, ‘In vivo advances in the 3D bioprinting of bone’ and ‘In vivo advances in the 3D bioprinting of OC tissue’ [2, 41–44]. Many cell types have been printed in combination with hydrogel materials and other biomolecules as tissue substitutes [45]. Through the manipulation of printing parameters, biomaterial properties and the use of bioactive molecules, the behaviour of a single cell and/or a whole colony can be modulated, resulting in cell-instructive scaffolds [46]. Additional cues, such as stiffness, microarchitecture and, most crucially, the zonal topography modulated by 3D printing, can provide biophysical signals to instruct cells towards tissue-specific differentiation and ECM formation [47].

**Fig. 2:**
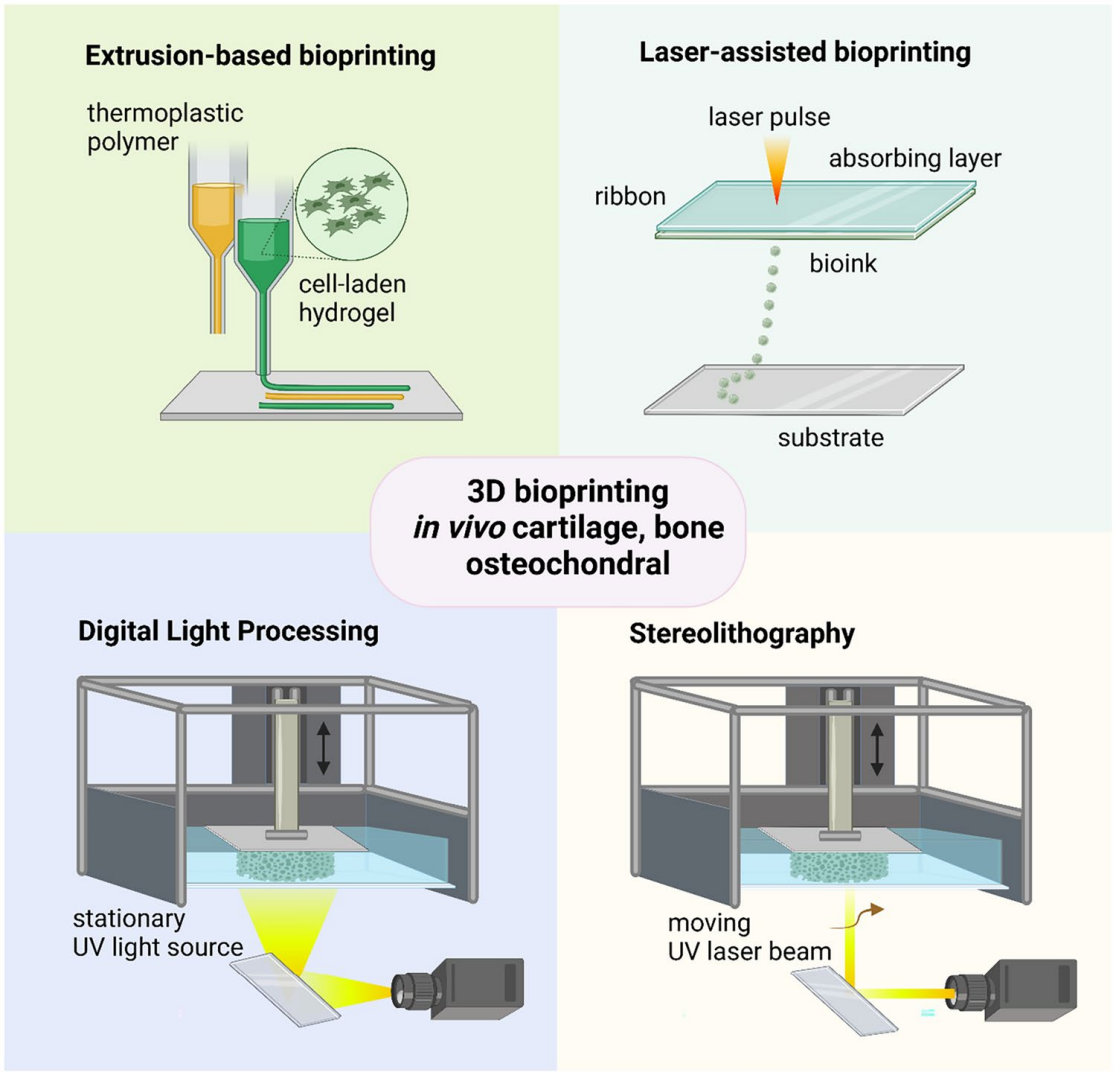
Schematic illustration of the four main 3D bioprinting techniques utilized in the fabrication of scaffolds for the in vivo regeneration of cartilage, bone and OC tissues

Bioprinted scaffolds for in vivo bone, cartilage and OC tissue regeneration can be fabricated using different methods. The description of all 3D bioprinting techniques is out of the main scope of this review and can be conveniently found in recently published works [1, 48, 49]. Therefore, in this section, we focused only on those fabrication processes used in the papers that were included in this review according to the inclusion criteria described in ‘Introduction’. As shown in Fig. 2, in this section, we describe extrusion-based bioprinting, laser-assisted bioprinting (LAB), digital light processing (DLP) and stereolithography (SLA).

Extrusion printing involves loading the material in a cartridge and then extruding it through a nozzle, with a diameter in the 0.1–1 mm range, to dispense strands following a layer-by-layer pattern to fabricate the bioscaffold (Fig. 2) [22]. The extrusion system can be pneumatic, piston driven or screw driven, while a heating element may be added to plasticize/melt thermoplastic polymers and optimize filament viscosity [50]. For instance, porous scaffolds composed of a cell/drug-loaded hydrogel, printed within the microchannels generated by extruding a primary thermoplastic ink, have been developed for musculoskeletal tissue applications [14, 51, 52]. In this approach, the primary ink provides adequate mechanical support [51–53], whereas the hydrogel, printed under mild conditions, is suitable for encapsulation of cells and biomolecules [2]. Hydrogels made of alginate (Alg), gelatin (GeL) and hyaluronic acid (HA), or their chemically modified derivatives, are among the most used bioinks for 3D bioprinting of cell-laden tissue constructs [54]. In extrusion 3D bioprinting, the bioactivity of encapsulated cells and active factors is still an important issue as processing conditions, namely the shear forces during hydrogel extrusion, the temperature/solvent required for thermoplastic polymer printing and the light exposure, may affect the functions of cells and biomolecules [55, 56].

LAB is a direct writing process based on the laser-induced forward transfer technology and uses three main components [57]: a pulsed laser source, a ribbon and a receiving substrate (Fig. 2). In order to achieve a pulse energy accumulation with 1–20 J per pulse, nanosecond lasers with ultraviolet (UV) wavelengths like those of excimer laser at 193 nm and 248 nm, or near-UV wavelengths at 1064 nm, are typically used as energy sources [58, 59]. The ribbon is a multilayer component including a transparent glass, a thin layer of laser-absorbing metal such as gold or titanium and a suspended layer of bioink usually made of hydrogels, containing cells and/or bioactive factors. The metal layer on top of the hydrogel is vaporized when the laser beam pulses for a certain amount of time focused on the ribbon. This results in a high-pressure bubble that ejects the bioink droplets onto the receiving substrate. LAB provides a higher printing resolution compared to nozzle-based bioprinting, and the resolution depends on different factors such as the thickness of the bioink layer, the viscosity and surface tension of the bioink, the wettability of the substrate, the laser wavelength and power and the air gap between the ribbon and the substrate. The main advantages of LAB are as follows: the high printing resolution, down to the micron level [60]; the possibility to process highly viscous bioinks (in a range of 1–8000 mPa∙s) [61, 62] and high-cell density bioinks necessary for development of blood vessels [63]; the safe printing conditions that ensure high cell viability [64]; and, ultimately, the possibility to be implemented for in situ printing [65, 66].

DLP and SLA belong to the category of vat polymerization techniques, as they use a photo-curable liquid bioresin that is radiated by its specific curing wavelength following a CAD pattern to achieve the final 3D construct. DLP and SLA enable the incorporation of living cells and biomolecules towards the fabrication of patient-specific implants and TE scaffolds [5, 6]. The selection of laser source and exposure time are the key factors to optimize the quality and resolution of photo-polymerized object, as well as to preserve the viability and activity of encapsulated cells and biomolecules. DLP typically uses a stationary UV light source from a projector, which cures the entire layer at once, whereas SLA utilizes a moving UV laser beam, which cures the layer by moving from point to point according to a certain pattern (Fig. 2). For these reasons, even if SLA provides a highly defined replica of the virtual model, it is a time-consuming process and, therefore, DLP may be preferable when there is the need to reduce fabrication time.

## In vivo advances in the 3D bioprinting of cartilage

### Cellularized biomaterials

The scarce presence of cells in cartilage is one of the reasons for the limited self-healing capacity of this tissue [67]. Indeed, the presence of metabolically active cells is crucial for the ECM biosynthesis and, therefore, for the formation of a functional tissue. From a tissue engineering point of view, one of the first points to address is the identification of the most appropriate cell type to use.

According to our search (Table 1), primary CCs and mesenchymal stem cells (MSCs) are among the most used cellular types for in vivo investigation of bioprinted scaffolds. Importantly, several studies employ a mixture of MSCs and CCs [68–72]. Indeed, MSCs can contribute to cartilage formation not only by differentiating towards CCs, but also by playing a trophic role on CCs. This effect can be mediated by the secretion of active factors (e.g. cytokines and growth factors), as well as by a direct cell–cell contact with CCs [68]. Moreover, the use of a MSC/CC co-culture system allows to reduce the number of CCs, which is a relevant advantage considering the limited sources of CCs in clinical settings [68]. Finally, MSCs usually do not undergo dedifferentiation, a process that is often observed for CCs. Accordingly, the research group of Lars Kölby demonstrated that the use of the MSC/CC co-culture in vivo may be preferable to the mono-culture in a bioink made of nanocellulose (NC) and Alg (Table 1) [68–71]. For example, Möller et al. [68] and Apelgren et al. [69] employed a mixture of human bone marrow–derived MSCs (BMSCs) and human nasal CCs in the above-mentioned bioink. They observed a higher cell proliferation and a more pronounced deposition of CoL type II and glycosaminoglycans in the co-culture group compared to mono-culture, in an in vivo subcutaneous mouse model [68, 69].

**Table 1:** Material composition, cell type and/or active molecule, animal model and main outcome of in vivo studies on 3D bioprinted cartilage constructs

**Material**	**3D (bio)printing technique**	**Cell type**	**Active molecule**	**Animal model**	**In vivo outcome**	**Ref.**
NC Alg	Extrusion	Human BMSCs Human nasal CCs	–	Subcutaneous, mouse	Structural integrity over 60 days Beneficial effect of co-culture on cartilage formation	[68]
NC Alg	Extrusion	Human BMSCs Human nasal CCs	–	Subcutaneous, mouse	Beneficial effect of co-culture on cartilage formation	[69]
NC Alg	Extrusion	Human MSCs Human nasal CCs	–	Subcutaneous, mouse	Good tissue integration	[70]
NC Alg	Extrusion	Human nasal CCs	–	Subcutaneous, mouse	Structural integrity over 60 days Cell proliferation and deposition of cartilage markers	[78]
NC Alg	Extrusion	Human nasal CCs Human BMSCs SVF-derived stem cells	–	Subcutaneous, mouse	Long-term safety, construct integrity, cartilage formation	[71]
NC Alg	Extrusion	Human nasal CCs	–	Subcutaneous, mouse	Cell proliferation, construct integrity, blood vessel ingrowth	[79]
GelMA	Extrusion	Rabbit BMSCs (standard MSCs vs MSCs with upregulated microRNA-410)	–	Orthotopic, rabbit (distal femoral condyle defect)	Better cartilage repair in the group of MSCs with upregulated microRNA-410	[75]
GelMA HAMA	Extrusion	Sheep MSCs	–	Orthotopic, sheep (lateral and medial femoral condyle defects)	Feasibility of in situ 3D bioprinting by a hand-held device Early formation of hyaline-like cartilage Lack of lateral integration	[76]
CoL type I	Extrusion	Rat xiphoid CCs	–	Subcutaneous, rat	Premature resorption Inflammation No cartilage formation	[84]
SFMA	DLP	Human nasal CCs Rabbit auricular CCs	–	Subcutaneous, mouse Orthotopic, rabbit (partial trachea defect)	Cartilage formation	[83]
SF HPCMA	Extrusion	Rabbit BMSCs	–	Orthotopic, rabbit (patellar groove defect)	Better cartilage regeneration for the SF-HPCMA group compared to the SF group	[74]
PCL CoL	Dual extrusion (hydrogel extrusion + PCL melt extrusion)	Rabbit articular CCs	–	Orthotopic, rabbit (femoral condyle defect)	Superiority of porous CoL hydrogel over PCL-reinforced CoL hydrogel regarding cartilage-like tissue formation Inflammation caused by PCL	[82]
PCL Fibrin	Dual extrusion (hydrogel extrusion + PCL melt extrusion)	Rabbit BMSCs	–	Orthotopic, rabbit (lateral and medial femoral condyle defects)	Anisotropic cartilage regeneration by scaffolds with pore size–dependent, bottom-up gradient	[73]
PCL Alg	Dual extrusion (hydrogel extrusion + PCL melt extrusion)	Mouse chondrogenic cells	–	Subcutaneous, mouse	Deposition of cartilage-specific ECM Feasibility of non-invasive assessment of scaffolds in vivo	[86]
PCL Alg	Dual extrusion (hydrogel extrusion + PCL melt extrusion)	Rabbit auricular CCs	–	Orthotopic, rabbit (auricular defect)	Superiority of PCL/hydrogel scaffolds over plain PCL scaffolds regarding cartilage-like tissue formation	[87]
PCL CoL	Dual-rotation extrusion (hydrogel extrusion + PCL melt extrusion)	Human nasal CCs Human nasal turbinate stem cells	–	Subcutaneous, mouse	Cartilage formation PCL prevents scaffold resorption	[72]
PCL dECM GelMA	Dual extrusion (hydrogel extrusion + PCL melt extrusion)	–	Aptamer HM69 TGF-β3	Orthotopic, rabbit (femoral defect)	Cell recruitment, cell differentiation, cartilage regeneration	[89]
PCL dECM GelMA PLGA (µPs)	Dual extrusion (hydrogel extrusion + PCL melt extrusion)	–	TGF-β3	Orthotopic, sheep (femoral condyle defect)	Cartilage regeneration, zone-dependent CoL orientation of regenerated cartilage	[90]
dECM	Extrusion (low-temperature deposition manufacturing (LDM))	–	Growth differentiation factor 5 (GDF-5)	Orthotopic, rabbit (femoral defect)	Better cartilage regeneration and tissue integration with the surrounding tissue for the GDF-5 group compared to the GDF-5-free group	[99]
PCL GeL Fibrinogen HA PLGA (µPs)	Dual extrusion (hydrogel extrusion + PCL melt extrusion)	Rabbit BMSCs	TGF-β3 BMP4	Orthotopic, rabbit (trochlear groove defect)	Anisotropic cartilage regeneration and cell phenotype by depth-dependent pore size distribution and spatio-temporal release of growth factors	[91]
PCL GeL Fibrinogen HA Glycerol PLGA (µPs)	Dual extrusion (hydrogel extrusion + PCL melt extrusion)	Rabbit BMSC	GDF-5	Orthotopic, rabbit (femoral defect)	Cartilage regeneration, long-term chondroprotection	[97]
PCL GeL Fibrinogen HA Glycerol PLGA (µPs)	Dual extrusion (hydrogel extrusion + PCL melt extrusion)	Goat BMSCs	TGF-β3 CTGF	Orthotopic, goat (total meniscectomy)	Anisotropic cartilage regeneration and cell phenotype by spatio-temporal release of growth factors	[92]
Alg Alg sulphate GelMA	Extrusion	Porcine BMSCs	TGF-β3	Subcutaneous, mouse	Cartilage-like tissue formation	[93]
SF dECM	Extrusion	Rabbit BMSCs	TGF-β3	Subcutaneous, mouse	Chondrogenesis and cartilage-like tissue formation	[94]
GelMA	Extrusion	Rat BMSCs	Platelet-rich plasma	Subcutaneous, mouse	Chondrogenesis and cartilage-like tissue formation	[95]
GelMA HAMA CSMA	Extrusion	Rat synovium-derived MSCs	TGF-β1	Orthotopic, rat (trochlear groove defect)	Better cartilage regeneration and tissue integration for the TGF-β1 group compared to the TGF-β1-free group	[96]

Overall, the cell density used for the cellularization of 3D bioprinted constructs, including CCs or MSCs or a mixture of the two, usually ranges between 1 × 10^6^ and 2 × 10^7^ cells/mL, with 1 × 10^7^ cells/mL being the most common cell density used. Importantly, when a mixture of MSCs and CCs is used, a much larger portion of MSCs is employed compared to CCs, which significantly reduces the number of needed CCs. In this case, the MSC/CC ratio typically ranges between 4:1 and 3:1, with the ratio 4:1 being the most used [68–72].

Regarding the cell source, when using stem cells in co-culture with CCs or in mono-culture, most of the papers report positive outcomes in cartilage formation when using BMSCs in 3D bioprinted constructs implanted in subcutaneous mouse models and in orthotopic rabbit models [68–71, 73–75]. As an alternative, Di Bella et al. [76] used adipose-derived stem cells (ADSCs) isolated from the infra-patellar fat pad in a pilot study based on the orthotopic implantation in sheep. The use of adipose tissue as a source of stem cells has been also explored by Apelgren et al. [71], who used the lipoaspirate called stromal vascular fraction (SVF) as a source of stem cells. In a comparative study, the authors observed that SVF-derived stem cells had a similar trophic effect on the proliferation of CCs compared to BMSCs, in a long-term (10-month) subcutaneous mouse model. From the perspective of clinical translation, the use of SVF may be relevant because the cell harvesting process is relatively simple and can lead to a high number of cells. Moreover, SVF-derived cells do not need expansion or other in vitro manipulations, which could potentially facilitate their regulatory approval [71].

Concerning the used biomaterials, all studies report the use of hydrogels based on natural polymers, such as NC, Alg, CoL, GeL, HA, silk fibroin (SF) or partially modified natural polymers, mainly GeL methacrylamide (GelMA) and methacrylate HA (HAMA). This is because hydrogels are water-rich matrices able to provide a friendly environment for cell proliferation and, in some cases, to offer receptor-mediated cell adhesion, depending on the used polymer. For example, GeL and CoL can establish cell interactions mediated by the arginylglycylaspartic acid (RGD) peptide motif, whereas HA can interact with cells via the CD44 receptors. Moreover, hydrogels usually present a shear-thinning/fast recovery behaviour at cell-friendly temperatures, which allows the material to maintain the shape of the generated pattern after printing [77]. Although hydrogels made of synthetic polymers or a mixture of synthetic and natural polymers are reported for cartilage 3D bioprinting in vitro, none of them appears when limiting the search to in vivo studies of cellularized 3D bioprinted cartilage constructs. This points out some intrinsic limitations of synthetic polymers compared to natural polymers, such as the absence of active sites for cell interaction, as well as time-consuming synthetic steps for novel polymers synthesized at a bench scale.

Among the various biomaterials under investigation, a bioink made of NC and Alg has been reported to support in vivo cartilage formation in a subcutaneous model [68–71, 78, 79]. NC is a sustainable, biocompatible material with good mechanical properties [80]. Interestingly, bacterial NC fibrils have a width of approximately 100 nm, which makes them similar to CoL fibrils [81]. When properly blended, NC confers shear thinning properties, whereas Alg is used to promote a CaCl_2_-mediated physical gelation after printing [68]. Other hydrogel systems rely on the chemical cross-linking of CoL mediated by genipin [82] or on the UV-mediated cross-linking of methacrylated polymers, such as GelMA, HAMA [75, 76], hydroxypropyl cellulose methacrylate (HPCMA) [74] or methacrylated SF (SFMA) [83]. In contrast, Isaeva et al. [84] reported the 3D bioprinting of a cell-laden hydrogel based on high-concentration CoL without chemical cross-linking. However, in this case, a premature resorption of the scaffold was observed in a subcutaneous mouse model.

Although hydrogels are widely investigated for cartilage 3D bioprinting, they often do not provide the mechanical properties required to withstand the load that cartilage normally bears under physiologic conditions [85]. The mechanical challenge is evident especially in the case of the orthotopic implantation of scaffolds in medium- and large-sized animals, which better simulates the clinical application compared to the subcutaneous implantation in small animals. To address this challenge, several groups have designed 3D printed constructs based on mechanically robust PCL fibres. PCL is a slow-degrading polyester featuring thermoplastic properties that enable it to be 3D printed using melt extrusion. For these reasons, PCL has been widely investigated in cartilage regeneration. Usually, hybrid PCL/hydrogel scaffolds are fabricated by printing an alternating filament of PCL and cell-laden hydrogel [72, 73, 82, 86, 87]. Importantly, this approach combines the mechanical robustness of a PCL mesh and the cell-friendly environment of the hydrogel with the possibility of tuning scaffold porosity and distributing cells in a zone-dependent fashion. For example, Sun et al. [73] fabricated gradient scaffolds where different pore sizes were used in the superficial layer and in the deep layer to induce anisotropic cartilage regeneration. This pore size gradient was obtained by varying the fibre spacing during printing (150 µm in the superficial layer and 750 µm in the deep layer). The used scaffold design induced a zone-dependent gene expression and cellular phenotype in vivo, resembling the anisotropic structure of human cartilage, and induced microvascularization in the deep layer [73]. Overall, several authors report positive outcomes in terms of in vivo cartilage formation by using the PCL/hydrogel double printing approach [72, 73, 86, 87]. However, some open questions about the use of PCL remain, especially regarding whether the tough, long-lasting micro-sized PCL fibres are fully beneficial in vivo. Of note, Koo et al. [82] reported inflammatory reaction of a CoL/PCL double-printed scaffold, which was attributed to the excessive strength of PCL. The same authors also reported an overall better performance of PCL-free, 3D bioprinted CoL scaffolds in terms of cartilage formation in an orthotopic rabbit model [82].

### Acellular biomaterials functionalized with active factors

Cell-free scaffold implantation may overcome drawbacks associated with cell-based cartilage tissue engineering strategies, such as the high cost and the complex procedures for cell harvest, expansion and handling as well as the optimization of cell density and spatial distribution. When using acellular scaffolds, the recruitment, proliferation and differentiation of endogenous cells become crucial aspects [88]. To support such cascade, biomaterials can be made by biomimicking materials, such as decellularized ECM (dECM), and can be functionalized with active factors. Yang et al. [89] described a novel scaffold based on the double printing of mechanically reinforcing PCL and a hydrogel made of dECM and GelMA, enriched with two active factors, namely the aptamer HM69 and the transforming growth factor (TGF)-β3. In this system, the aptamer acted as a recruiting agent for endogenous MSCs and the TGF-β3 as a chondrogenic factor in vivo, leading to cartilage-like tissue formation.

When functionalizing scaffolds with active factors, clearance and inactivation of these factors in the joint environment represent a major hurdle [14]. Chemical immobilization strategies of active factors into 3D bioprinted scaffolds have been proposed to slow down the active factor clearance and to enhance cartilage regeneration in vivo [89]. Alternatively, the encapsulation of active factors into delivery systems may be used to preserve protein activity and to achieve a better control over its spatio-temporal release. Yang et al. [90] encapsulated TGF-β3 into poly(lactic-*co*-glycolic) acid (PLGA) microparticles (µPs) and used this delivery system to functionalize a 3D bioprinted scaffold based on the double printing of PCL and a hydrogel, similar to the one previously described. It is worth to note that the use of acellular scaffolds functionalized with active factors is much less investigated in vivo compared to cellularized scaffolds, while recent studies, such as those reported in the following section, demonstrated promising results by the synergic combination of cells and bioactive factors into bioprinted scaffolds.

### Synergies between cell therapy and drug delivery

Several studies demonstrated positive outcomes in terms of in vivo cartilage formation, when combining cellularization of 3D printed scaffolds with the controlled release of active factors. In all these papers, the authors typically report the use of BMSCs in combination with active factors able to support their chondrogenic differentiation. Depending on the different animal models used, cells are isolated from different species (e.g. rabbit, goat, porcine) and included in the bioink at a typical cell density ranging between 1 × 10^7^ and 2 × 10^7^ cells/mL [91–95]. In contrast, Sang et al. [96] proposed synovium-derived MSCs (used at a lower cell density of 1 × 10^6^ cells/mL) as a promising alternative, thanks to their high ability of proliferation and chondrogenic differentiation, promoted by the high expression of CD44 and CD105 receptors of these cells.

TGF-β3 is the most used active factor for cartilage repair, either alone [93, 94] or in combination with other growth factors, such as bone morphogenic protein (BMP), in zonally organized 3D printed scaffolds [91, 92]. In the latter approach, different factors are distributed in a zone-dependent fashion to obtain a zone-dependent ECM deposition resembling that of human hyaline cartilage or meniscus. For example, Sun et al. [91] developed a dual-factor releasing scaffold by 3D bioprinting a cell-laden hydrogel functionalized with BMP4 in the deepest layer and with TGF-β3 in the middle and top layers. The authors observed a regionally dependent cartilage regeneration in vivo, demonstrated by the immunostaining of zone-specific markers (e.g. superficial distribution of proteoglycan 4, gradient expression of CoL type II and preferential localization of CoL type X in the deep layer) [91]. A similar approach was also applied to meniscus regeneration by 3D printing a cell-laden hydrogel enriched with connective tissue growth factor (CTGF) in the outer zone and with TGF-β3 in the inner zone [92]. The different spatio-temporal releases of these two factors supported anisotropic meniscus regeneration (e.g. zonal expression of CoL types I and II) and zonal cell phenotype in vivo.

Three different drug delivery strategies can be identified for the immobilization and controlled release of active factors in 3D bioprinted scaffolds for in vivo cartilage regeneration, namely (i) encapsulation into PLGA µPs [91, 92, 97], (ii) interactions between the heparin binding sites of growth factors and sulphated polymers [93] and (iii) application of growth factors onto the scaffolds after 3D bioprinting [95]. Among them, PLGA microencapsulation of active factors seems to be the most used approach, as PLGA is one of the most investigated polymers for the controlled delivery of therapeutic proteins. Indeed, in PLGA-based drug delivery systems, the release kinetics of growth factors can be properly tuned by changing PLGA characteristics such as the lactic acid/glycolic acid ratio, molecular weight, capping group as well as particle size and porosity [98]. Moreover, PLGA µPs may protect growth factors during scaffold fabrication (e.g. 3D printing, cross-linking) and post-fabrication processing (e.g. sterilization). Importantly, the control over the spatial distribution of growth factors via 3D bioprinting and the control over the release kinetics offered by PLGA microencapsulation offer a relevant opportunity to achieve a spatio-temporally controlled release of biomolecules.

**Fig. 3:**
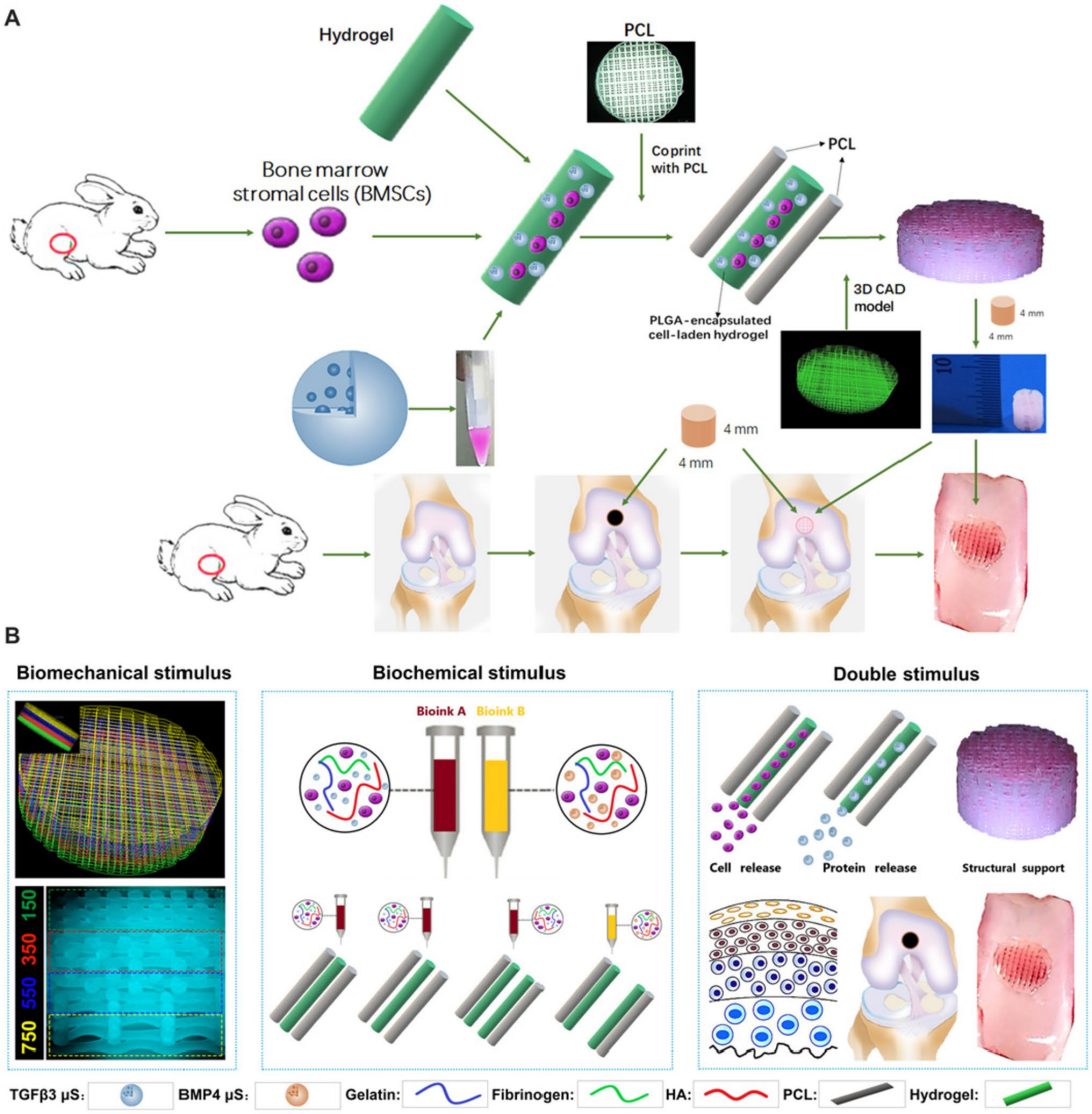
**A**, **B** Representation of a 3D bioprinted zonal scaffold as reported by Sun et al. [91]. The scaffold is composed by a cell-laden bioink made of GeL, fibrinogen and HA, reinforced by 3D printed PCL. Zonality is induced by varying the line spacing from 150 µm (top) to 750 µm (bottom) and by distributing TGF-β3-loaded PLGA µPs in the top and middle layers and BMP4-loaded µPs in the deepest layer. Reproduced from Sun et al. [91] without any modification

Biomaterials are usually composed of a cell/active factor–loaded hydrogel bioprinted solely or within the microchannels of printed PCL filaments, possibly with zone-dependent porosity [91, 92, 97]. For example, by gradually varying the filament spacing from 150 to 750 µm (from top to bottom), Sun et al. [91] fabricated gradient scaffolds with a zone-dependent porosity which supported anisotropic cartilage regeneration in vivo, with a favourable chondrogenic differentiation in the top layer and guiding a preferential vessel growth in the deeper layers. Figure 3 outlines this approach which also relied on the combined use of BMSCs and PLGA-microencapsulated BMP4 and TGF-β3.

Among the hydrogel-forming building blocks, GeL is the most used, due to its several favourable properties, namely (i) biodegradability, (ii) cell binding sites, (iii) thermosensitive and shear thinning properties which make GeL-based hydrogels 3D printable over a broad range of polymer concentration and (iv) chemical cross-linking when using GelMA. GeL-based bioinks are usually made by physically mixing GeL or GelMA with other components to achieve optimal mechanical properties [91–93, 97]. For example, GelMA has been combined with other methacrylated polysaccharides, such as HAMA and chondroitin sulphate methacrylate (CSMA), or with Alg and Alg sulphate to form interpenetrating networks by combining UV-mediated chemical cross-linking with Ca^2+^ ion–mediated physical gelation [93, 96].

## In vivo advances in the 3D bioprinting of bone

### Cellularized biomaterials

Cellular-based approaches in bone TE primarily target the early stages of bone repair when the recruitment of skeletal progenitor cells may be impaired [100]. Different cell sources, types and differentiation stages have been successfully used to support bone regeneration. Although bone marrow–derived osteoprogenitors were the first cell type used in bone TE, to date, there is a growing interest also in multipotent stem cells which are under increased scrutiny for TE applications [101]. Regarding bone repair and regeneration, the most used cells are MSCs, embryonic stem cells (ESCs), induced pluripotent stem cells (iPSCs), ADSCs as well as human umbilical vein endothelial cells (HUVECs) to improve vascularization and formation of tube-like structures resembling early capillaries (Table 2) [102, 103].

**Table 2:** Material composition, cell type and/or active molecule, animal model and main outcome of in vivo studies on 3D bioprinted bone constructs

**Material**	**3D (bio)printing technique**	**Cell type**	**Active molecule**	**Animal model**	**In vivo outcome**	**Ref.**
Fibrin GelMA HA Glycerol Fibrinogen	Extrusion + drop-on-demand	HUVECs Human ADSCs	–	Subcutaneous, mouse	Blood vessel formation Calcified bone matrix	[117]
CoL β-TCP	Extrusion	Human ADSCs HUVECs	–	Orthotopic, mouse (posterolateral lumbar spinal fusion)	New bone formation and angiogenesis	[118]
GelMA	Extrusion	Rat MSCs	–	Orthotopic, rat (distal femoral condyle)	Evident osteoconductive features	[108]
GelMA PLA-PEG-PLA	Extrusion	BMSCs ECs	–	Orthotopic, rat (calvarial defect)	New bone formation Control of in situ angiogenesis	[120]
CoL PCL	Dual extrusion (hydrogel extrusion + PCL melt extrusion)	Human nasal turbinate–derived MSCs Human BMSCs	–	Subcutaneous, mouse Orthotopic, rat (tibial defect)	Greater osteoinductive and osteoconductive potential of human nasal turbinate–derived MSCs than human BMSCs	[113]
GeL Alg Laponite nPs	Extrusion	Rat BMSCs	–	Orthotopic, rat (calvarial defect)	High bone healing capability	[105]
PCL Fibrinogen GeL HA Glycerol	Dual extrusion (hydrogel extrusion + PCL melt extrusion)	Human BMSCs	–	Subcutaneous, rat	Good vascularization and mineralization	[107]
CoL Hap nPs	In situ direct laser-assisted bioprinting (LAB)	Mouse bone marrow mesenchymal stromal cells	–	Orthotopic, mouse (calvarial defect)	Different cellular arrangements impact on bone tissue regeneration	[65]
SilMA GelMA Alg CoL microgels	Extrusion	Rat BMSCs	–	Subcutaneous, rat Orthotopic, rat (skull defect)	Microgels construct exhibited higher bone formation compared to neat construct	[104]
Gel Alg Laponite nPs	Extrusion	Rat BMSCs	–	Subcutaneous, rat (muscle pouch) Orthotopic, rat (cranial defect)	Protection of cells during printing; Activation of the PI3K/AKT signalling pathway	[106]
Fibrin PCL	Dual extrusion (hydrogel extrusion + PCL melt extrusion)	HUVECs Human MSCs	–	Subcutaneous, rat Orthotopic, rat (cranial defect)	Mechanical strength comparable to bone Mimicking of native bone cell pattern	[119]
Laponite nPs Hap nPs Alg Methylcellulose PCL	Dual extrusion (hydrogel extrusion + PCL melt extrusion)	–	VEGF BMP2	Subcutaneous, mouse Orthotopic, rat (segmental defect)	Spatially defined BMP2 release kinetics, accelerated bone healing	[140]
PCL TCP HAMA GelMA	Dual extrusion (hydrogel extrusion + PCL melt extrusion)		RSV Strontium ranelate	Orthotopic, rat (mandibular defect)	Improved bone formation	[138]
GeL PVA HA Hap/PCL nPs	Dual extrusion (hydrogel extrusion + PCL melt extrusion)		DX	Orthotopic, rabbit (proximal tibia defect)	Superior osteogenic performance of low-dose DX-integrated scaffolds	[135]
PLGA/TCP	Low-temperature extrusion		ICT Human fetal MSCs Secretome	Orthotopic, rat (osteoporotic bone defect)	Enhanced regenerative capacity of osteoporotic bone	[143]
Alg GG CPC	Dual extrusion (extrusion of CPC + extrusion of Alg)		VEGF	Orthotopic, rat (segmental defect in the femur diaphysis)	Enhanced vascularization in the defect region	[141]
GelMA Hap	Extrusion	BMSCs	TDN CLI	Orthotopic, rat (bone defect on the lateral condyle)	Excellent biocompatibility, osteogenic and antimicrobial activity	[166]
Alg GeL Fibrin PCL Hap	Dual extrusion (hydrogel extrusion + PCL melt extrusion)	HUVECs hBMSCs	RGD	Subcutaneous, mouse Orthotopic, rat (femoral defect)	Support of vessel formation	[176]
Chitosan nPs β-Glycerophosphate Chitosan CoL CoL sponge Hap nPs	Extrusion intraoperative bioprinting (IOB)	Rat BMSCs	Plasmid DNAs PDGF-B BMP2	Orthotopic, rat (calvarial defects)	Significant amount of newly formed mineralized bone	[148]
GelMA GGMA	Extrusion	HUVECs Rat BMSCs	DFO	Subcutaneous, mouse Orthotopic, mouse (intracranial defect)	Improved vascularization in subcutaneous implant Activation of HIF1-α in the intracranial implant	[160]
CoL	Extrusion	Human ADSCs	cBMC (chicken bone marrow cells)	Orthotopic, rat (mastoid obliteration)	Ability of cBMC to induce bone regeneration	[177]
CoL	In situ LAB	SCAPs HUVECs	VEGF	Orthotopic, mouse (calvarial defect)	Generation of microvascular networks with defined configurations into critical-sized bone defects	[66]
Sodium Alg GeL Hap nPs	Extrusion	Human PDLSCs	SDSSD	Subcutaneous, mice Orthotopic, mouse (skull defect)	SDSSD promoted bone formation by binding to G protein–coupled receptors and regulating the AKT signalling pathway	[150]
GelMA	Extrusion	Mouse BMSCs Murine umbilical vein ECs	ATP	Orthotopic, rat (cranial defect)	Nano-ATP composite hydrogels improved bone regeneration and promoted angiogenesis	[173]
GeL GelMA 4-arm acrylated PEG	Extrusion	Rat BMSCs RAW264.7	BMP4	Orthotopic, rat (calvarial defect)	Reduction of pro-inflammatory factors and stimulation of osteogenic differentiation	[170]
PLGA PEG Pluronic 127	Extrusion	Human MSCs	GET-RUNX2	Orthotopic, mouse (distal femur defect)	Mechanically strong ‘cancellous bone-like’ printable implants High-density bone development	[149]
GelMA AlgMA Laponite	Extrusion	Rat BMSCs	NGF	Subcutaneous, rat Orthotopic, rat (cranial defect)	Differentiation of BMSCs and formation of a neural network	[168]
Gel Fibrinogen HA Glycerol Pluronic F-127 Thrombin PCL	Dual extrusion (hydrogel extrusion + PCL melt extrusion)	Rabbit BMSCs ECs	BMP4	Subcutaneous, mouse Orthotopic, rabbit (condyle defect)	3D microenvironment that promotes the formation of new blood vessels and new bone	[157]
GelMA HAMA PCL Mesoporous bioactive glass	Dual extrusion (hydrogel extrusion + PCL melt extrusion)	C3H10T1/2	DX	Subcutaneous, mouse	Efficacy of a DOX-containing scaffold in bone repair and prevention of infections	[156]

Several studies have shown that the use of MSCs in combination with hydrogels is an effective method for aiding the repair and regeneration of bone [65, 104–108]. This is due to their ability to be easily isolated and expanded by various tissue culture techniques and to differentiate into osteoblasts, among other cell types, thanks to their multipotent nature [109, 110]. Bone marrow has been the main source for MSC isolation as reported by several research groups [65, 105–108]. However, the use of BMSCs is limited by the invasive procedures necessary to extract them from the patient’s tissue, as well as by the low yield and decreased differentiation ability with the increase of donor age and cell passage number [111, 112]. Therefore, many studies have focused on finding new, valuable sources of stem cells. These sources should involve minimally invasive procedures and preserve cell potency [113]. For example, Yun et al. [113] investigated the possibility of using 3D printed human nasal turbinate–derived MSCs for in vivo bone regeneration. The authors showed that, if compared to human BMSC-laden CoL type I printed scaffold, the scaffold loaded with human nasal turbinate–derived MSCs had a greater osteoinductive and osteoconductive potential both in vitro and in vivo [113].

Tissue vascularization is the major limiting factor for fabricating human-scale bony tissues; thus, in vivo printing is limited to small-sized constructs that can allow nutrient/waste product diffusion [114]. Because of its central role in the supply of oxygen and nutrients to transplanted cells, and in the removal of waste products, scaffold vascularization is vital to both the ossification process and the subsequent bone production and remodelling [115]. In this context, the presence of endothelial cells (ECs) from different sources, such as HUVECs [116, 117], is a key aspect. Several studies employ a combination of HUVECS and MSCs from various sources to improve scaffold vascularization. For example, Rukavina et al. [117] generated 3D prevascularized bone tissue constructs by human ADSC and HUVEC extrusion–based bioprinting and drop‐on‐demand (DoD) bioprinting. Constructs implanted subcutaneously into immunodeficient mice showed proper vascularization after 12 days. Similarly, Kim et al. [118] developed a cell-laden scaffold based on CoL, β-tricalcium phosphate (TCP) and two cell types (human ADSCs and HUVECs), to promote bone regeneration and to develop an efficient vascular network by a crosstalk between HUVECs and human ADSCs. In another work, Piard et al. [119] reproduced the spatial pattern of HUVECs and human MSCs found in native osteons by the double bioprinting of a PCL matrix along a fibrin cell–laden hydrogel containing two separate osteogenic and vasculogenic cell populations to promote neovascularization.

While in most of these approaches a single bioink contains a physical mixture of the two cell types, some other authors used two different cell-specific bioinks for scaffold fabrication. For example, Shen et al. [120] bioprinted a BMSC-laden GelMA hydrogel to promote osteogenesis and a thermosensitive EC-laden PLA-polyethylene glycol (PEG)-PLA hydrogel to promote angiogenesis. This strategy enabled a more controlled distribution and seeding efficiency of the ECs and stimulated vascular network formation in vitro and in vivo.

The choice of the biomaterials is a key issue to achieve a proper implant integration, cell differentiation and functional tissue remodelling and to protect cells from damage during printing [121]. Natural hydrogels (e.g. CoL, GeL, HA, Alg, SF) and/or chemically modified hydrogels (e.g. GelMA, MeHA) are the most used scaffold biomaterials [122, 123]. There are strategies that go beyond the traditional direct inclusion of cells in the hydrogel. For example, Chai et al. [104] developed a strategy for cell encapsulation using microgels to enhance cell viability and reduce the cell damage caused by the shear stress generated during extrusion bioprinting. SFMA and GelMA were blended with cell-laden microgels to fabricate the 3D printed constructs. Results demonstrated that the microgel-containing construct showed better cell proliferation when compared with the SFMA/GelMA construct, and that it induced more bone formation in the defect area once implanted in vivo.

It has been widely reported that scaffolds’ mechanical properties affect cell functionality and their ability to form bone tissue, as cell differentiation and matrix biosynthesis can be enhanced by mechanical environmental stimuli and the presence of naturally occurring reinforcing materials [124]. For example, calcium phosphate cements (CPCs) have been proposed as promising bone substitutes able to mimic the bone mineral phase [125]. Hence, the use of composite materials based on hydrogels enriched with bioceramics is a common practice in bone TE to increase mechanical properties and stimulate osteogenesis by providing a calcium phosphate–rich environment [126–128]. Nanosilicate (NS), is a two-dimensional synthetic material composed of disk-shaped surface-charged nanoscale crystals that can form a physically cross-linked network with anionic, cationic and neutral polymers, featuring shear-thinning behaviour [105, 129]. Most notably, when embedded into hydrogels, NS particles promoted osteogenic differentiation of cells without growth factors. For example, Liu et al. [105] developed a functional and biomimetic nanocomposite bioink composed of rat BMSCs, NS, GeL and Alg for bone TE applications. Results indicated that rat BMSCs maintained good viability, and that NS stimulated cell proliferation up to 14 days in vitro. Moreover, in vivo testing, in a critical-sized calvarial defect of Sprague Dawley rats, showed that NS increased the osteoinductive potential of the cell-laden bioink. Similarly, Miao et al. [106] evaluated the effect of a laponite NS–containing hydrogel loaded with BMSCs on the in vivo rat cranial defect regeneration. They found that the addition of laponite increased the structural stability and mechanical properties of the hydrogel constructs and protected the encapsulated BMSCs during the printing process. Moreover, the bioink promoted BMSC osteogenic differentiation and induced ectopic bone formation without the addition of exogenous bone growth factors.

It is important to note that hydrogels, even if enriched with inorganic bioceramic fillers, do not provide the mechanical strength to withstand the in vivo stress that normally bone is subjected to [107, 113, 119, 120]. This limitation can be overcome by using mechanical reinforcements made of biocompatible and biodegradable synthetic polymers, such as PLA, polyglycolic acid (PGA), PLGA and PCL [130, 131]. For example, Pitacco et al. [107] developed a 3D bioprinted PCL-reinforced fibrin scaffold, featuring a central microchannel to improve nutrient transport and in vivo vascularisation, for large bone defect healing. Scaffolds made of PCL and a human BMSC-laden fibrin bioink supported vascularisation and endochondral bone formation when implanted into a critical defect of Wistar Han rats.

### Acellular biomaterials functionalized with active factors

**Fig. 4:**
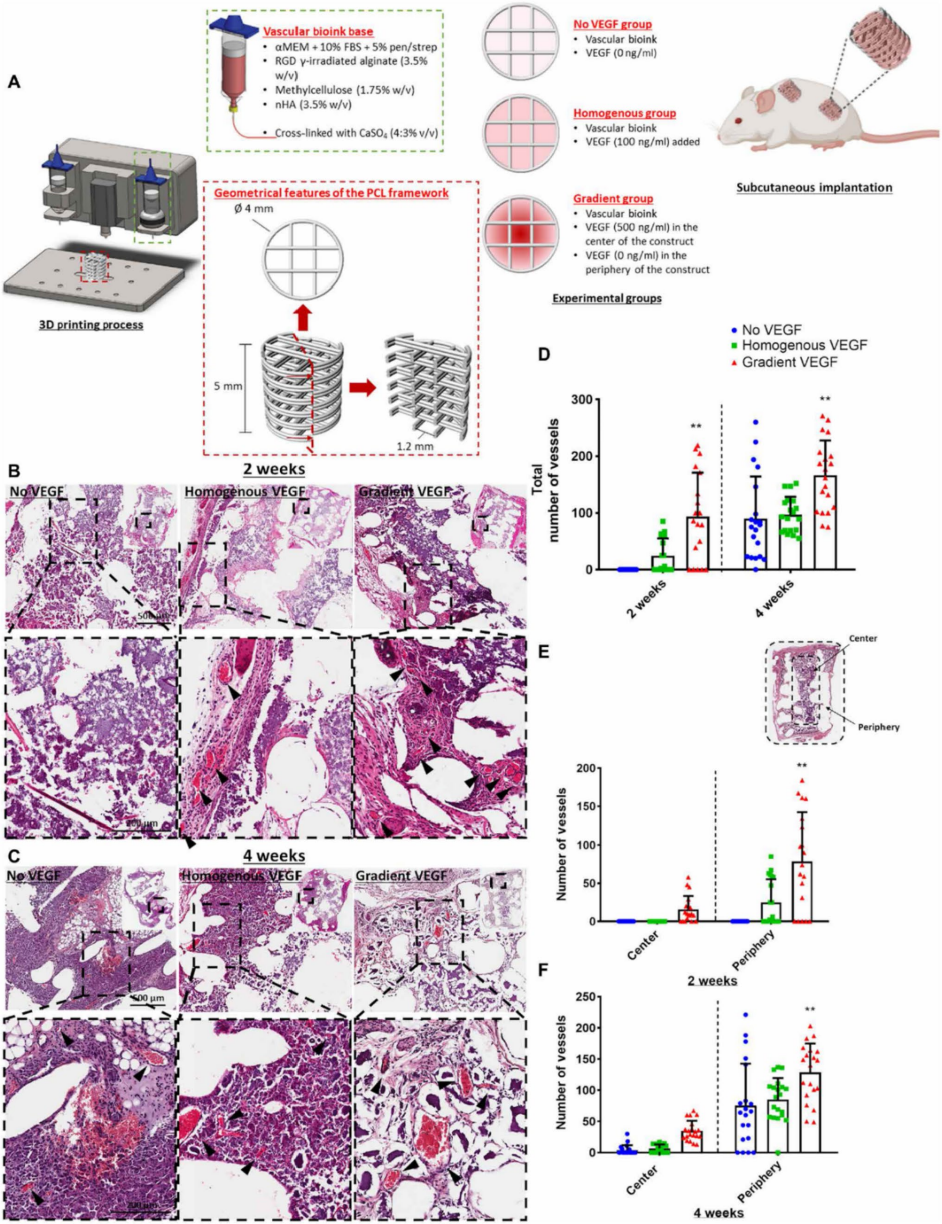
**A** Scheme of the 3D printed scaffold design and experimental groups of the work by Freeman et al. [140]. H&E-stained sections of the three experimental groups at **B** 2 weeks and **C** 4 weeks in vivo. **D** Total number of vessels of the experimental groups at 2 weeks and 4 weeks in vivo. Number of vessels present in the centre versus the periphery at **E** 2 weeks and **F** 4 weeks in vivo. Reproduced from ref. [140] without any modification

The continuous understanding of bone biology, the natural bone healing cascades and bone pathogenesis is boosting the design and creation of bone TE cell-free constructs able to deliver bioactive molecules to the injured site in a native-like manner [132]. This is because cell migration, adhesion, proliferation and differentiation occur in response to chemical cues present within the microenvironment, such as components of the ECM and morphogens or growth factors [133–138]. For instance, the inclusion of BMP2 and vascular endothelial growth factor (VEGF) into bone constructs may promote in vivo osteogenesis and vascularization, respectively [139, 140]. Accordingly, Freeman et al. [140] developed a range of nanoparticle-functionalized Alg bioinks to precisely control the spatial and temporal release of VEGF and BMP2 from 3D printed scaffolds. Three different constructs have been considered for the subcutaneous implantation as depicted in Fig. 4A: a construct with a homogenous VEGF distribution, a construct with a gradient VEGF distribution and a VEGF-free construct. The addition of laponite into the bioinks slowed down the release of VEGF and prolonged its spatial gradient for up to 14 days after printing, due to the strong attraction between the nanoparticles (nPs) and the growth factor. In this sense, the proposed biomaterial acted not only as a TE scaffold, but also as a controlled drug delivery system. As depicted in Fig. 4B, 2 weeks after implantation, histological analysis revealed the presence of vessels in the homogenous VEGF and gradient VEGF construct, whereas there were no vessels present in the VEGF-free construct. Of note, after 4 weeks (Fig. 4C), both the homogenous VEGF and the VEGF-free constructs showed mature vessel predominantly located in the periphery of the scaffold, while enhanced vascularization was observed in the gradient VEGF construct. Moreover, the combination of VEGF with BMP2 stimulated the formation of new bone into a segmental defect Fischer male rat model (Fig. 4) [140]. Similarly, Ahlfeld et al. [141] developed a biphasic scaffold based on CPC paste and VEGF-loaded Alg/gellan gum (GG) bioink. Their results demonstrated that the CPC component supported excellent osteoconductivity, whereas the local VEGF release stimulated EC proliferation and angiogenesis in vitro. In addition, in vivo experiments revealed the presence of new bone formation in a segmental bone defect.

The use of endogenous protective systems, e.g. secretome [142, 143], has been proposed as an alternative to the use of growth factors for bone TE. Secretome is defined as a set of secreted membrane-enclosed vesicles containing free nucleic acids and soluble proteins [144]. According to some works present in literature, secretome derived from human fetal MSCs promoted osteogenic differentiation of adult MSCs and enhanced bone consolidation in vivo [142]. From this perspective, Zhang et al. [143] proposed a combination of secretome derived from human fetal MSCs and from icaritin (ICT). ICT is an intestinal metabolite derived from the Chinese traditional medical plant *Epimedium* capable to promote the proliferation and differentiation of osteoblasts and enhance matrix calcification due to its estrogenic-like activity [145, 146]. This combination was able to improve the bioactive properties of a PLGA/TCP-based scaffold favouring the recruitment and differentiation of endogenous MSCs towards the osteoblast lineage in vitro [143]. Moreover, in vivo experiments in an osteoporotic bone defect rat model showed that the designed system promoted bone regeneration at the defect sites.

Other used osteogenic molecules in bone TE are resveratrol (RSV), simvastatin (SV) and doxycycline (DX) [135, 138]. For example, Zhang et al. [138] developed 3D printed scaffolds consisting of a PCL/β-TCP composite and a hydrogel-based bioink loaded with RSV and strontium ranelate that have been shown to promote bone formation by facilitating osteogenic differentiation and the release of angiogenic factors [138]. Results showed that the sustained release of RSV in combination with strontium ranelate promoted HUVEC angiogenesis induction and inhibited osteoclast activities. A similar approach was proposed by El-Habashy and co-workers [135], who developed a bioprinted scaffold made of a blend of GeL, polyvinyl alcohol (PVA) and HA and integrated with composite DX-loaded Hap/PCL nPs. Results confirmed the possibility to tune DX release over 28 days in vitro by a combined effect of freeze-drying process and nPs that act as a diffusion barrier. Moreover, the proposed nanocomposite scaffolds demonstrated their osteoconductivity, bioresorption, immune tolerance and bone regenerative potential in vivo when implanted in a proximal tibia model of New Zealand white rabbits [135].

### Synergies between cell therapy and drug delivery

The current research focuses on novel strategies that involve the synergistic combination of biomaterials, cells and active factors to create functional in vivo bone constructs providing faster and enhanced bone regeneration [100, 147]. Multifunctional bioactive constructs can be formulated by the combination of (i) hydrogels based on e.g. GeL, Alg, CoL and chitosan; (ii) growth factors, such as BMP2, BMP4, VEGF and platelet-derived growth factor (PDGF); and (iii) cells (e.g. BMSCs, human ADSCs and HUVECs). As native bone tissue development is controlled by the action of multiple growth factors acting in different sites and at different times, the success of this approach depends on the choice of growth factors, their combination as well as growth factors’ spatial and temporal gradients. For instance, the sequential release of PDGF and BMP2 supported more vascularized bone tissue formation compared to the simultaneous delivery of these two growth factors [148]. Moncal et al. [148] used a gene-activated matrix to control the delivery of PDGF-B from a rat BMSC-laden bioink and that of BMP2 from chitosan nPs. The bioink was directly bioprinted into critical-sized calvarial defects of Fischer white rats, showing a significant amount of newly formed mineralized bone when compared to the control group. Similarly, Awwad et al. [149] used the sustained release of recombinant glycosaminoglycan-binding enhanced transduction (GET) peptide-runt-related transcription factor 2 (RUNX2), encapsulated in PLGA µPs, to promote osteogenesis of human MSCs and bone formation in a mouse model.

Among all the biologically active molecules studied in literature, oligopeptides have been proposed as an alternative to growth factors due to their high bioavailability, lower synthesis cost and easier formulation [150, 151]. For example, Cai et al. [150]. combined the osteoblast-specific binding oligopeptide SDSSD with human periodontal ligament stem cells (PDLSCs) to develop a 3D bioscaffold and evaluated its physical and biological properties. The bioscaffold promoted the survival, proliferation and heterogeneous differentiation of human PDLSCs by activating the Akt signalling pathway that regulates many cellular functions such as metabolism, growth, proliferation, survival, transcription and protein synthesis [152]. Results showed that SDSSD increased bone formation in a subcutaneous mouse model and in a skull defect mouse model.

Another approach to overcome the constraints associated with the direct inclusion of growth factors is to genetically modify cells that can overexpress specific growth factors [153]. DX is a commonly used regulator of gene expression able to drive skeletal muscle–specific expression of the reverse tetracycline transactivator gene [154]. Moreover, DX can have the additional effect of inhibiting bacterial infection [155]. Exploiting these concepts, Wang et al. [156] developed a composite scaffold comprising a PCL/mesoporous bioactive glass/DX component and a bioink containing an engineered progenitor cell line (C3H10T1/2) capable of a DX-mediated release of BMP2. They demonstrated the efficacy of this system to repair infectious bone defects, guided by osteogenic differentiation and new bone formation.

The creation of well-defined patterns of osteon-mimetic scaffolds, including the hierarchical microchannel structure, is critical for the vascularization in bone defect repair [157, 158]. Sun et al. [157] designed a 3D microenvironment that mimics the native cell pattern in osteons and cortical bones. This was achieved by combining the osteogenic potential of BMP4 with ECs and BMSCs in a 3D printed scaffold with central medullary canals, peripheral Haversian canals and transverse Volkmann canals [157]. These constructs prompted the formation of new blood vessels and new bone, further accelerating the process of bone repair in vivo. A different strategy to improve vascularization was proposed by Kérourédan et al. [66], who used LAB to create specific patterns of HUVECs into mouse calvarial bone defects prefilled with CoL-VEGF-stem cells from the apical papilla (SCAPs)-seeded membrane. Two months after surgery, fluorescent vascular networks were found in experimental defects, suggesting a preservation of cell viability and confirming the ability of the proposed approach to generate microvascular structures.

Also when aiming at scaffold vascularization, small molecules may represent a valid alternative to growth factors and other proteins. For example, deferoxamine (DFO) is a small angiogenic agent under evaluation in studies on ischemia, wound healing and bone regeneration due to its capacity to upregulate the hypoxia-inducible factor 1-α (HIF1-α) signalling pathway, and it is involved in the process of angiogenesis and new bone formation [159]. Li et al. [160] combined DFO-loaded ethosomes (Eth) that are a particular type of liposomes with high deformability and high encapsulation efficiency, with GelMA/GG methacrylate hybrid hydrogels to modulate DFO release and to promote angiogenesis and bone regeneration. Results demonstrated vascularization and good biocompatibility of the scaffold in a subcutaneous model, as well as high bone formation in an intracranial model after 8 weeks.

In addition to blood supply deficiency, a reduction in the bone regenerating capacity is caused also by infections often occurring at the bone defect site [161]. Therefore, developing a biological scaffold material with effective antibacterial properties is a key issue. In this context, long-term local antibiotic therapy has obvious disadvantages, such as potential systemic toxicity, wound necrosis as well as antibiotic resistance [162]. Hence, alternatives have been suggested. Tetrahedral DNA nanostructures (TDNs), composed of four single-stranded DNA (ssDNA) molecules, possess good biocompatibility and a strong affinity for bacteria and mammalian cells showing anti-inflammatory and antioxidant properties [163, 164]. TDNs have been used as carriers for the delivery of ampicillin and reduced drug resistance by improving the movement of the drug across the cell membrane [165]. Starting from these results, Li et al. [166] proposed TDNs as a drug delivery system to enhance cell penetration and the antibacterial properties of clindamycin (CLI) that is a common antibiotic used to treat osteomyelitis [167]. TDN-CLI complexes were loaded in 3D bioprinted BMSC-laden GELMA/Hap hybrid scaffold. Results demonstrated that this system possessed excellent biocompatibility and antimicrobial activity and significantly improved the repair of infected bone defects in vivo.

Like the vascular network, also the neural network cannot be fully reconstructed by simply regulating osteogenic differentiation [168]. To date, the nervous system’s role in bone TE has been largely ignored even if early innervation is essential for the normal formation of ossification centre [169]. In this context, it is worth to mention the work by Li et al. [168], who developed a bioprinted construct made of GelMA and Alg methacrylate (AlgMA) hybrid hydrogel loaded with the nerve growth factor (NGF), laponite and BMSCs that simulated the ossification centre microenvironment. Authors demonstrated the synergic effect of NGF and Lap to the expression and secretion of calcitonin gene-related peptide, leading to the formation of a neural network and improved vascularization.

As reported in ‘Cellularized biomaterials’ in the section in vivo advances in the 3D bioprinting of bone, the addition of bioceramics to the bioink is a common practice in bone TE to increase mechanical properties and stimulate osteogenesis [126–128]. Importantly, reinforcing elements can also simultaneously act as delivery systems. For example, Sun et al. [170] developed a bioink comprising GeL, GelMA and 4-arm poly(ethylene glycol) acrylate containing BMSCs, RAW264.7 macrophages and BMP4-loaded mesoporous silica nPs. The nPs improved both scaffold mechanical strength and BMP4 release. Furthermore, in vivo results showed that the composite scaffold improved diabetic bone repair, owing to the direct effects of BMP4 on promoting osteogenesis of BMSC scaffolds.

Similar to NS, also nanoclays have drawn increasing attention for the fabrication of biomedical materials for bone TE [171–174]. Attapulgite (ATP) (Al_2_Mg_2_Si_8_O_20_(OH)_2_(OH_2_)_4_), a naturally abundant nanoscale hydrated magnesium-rich clay mineral with a rod-like crystalline structure, has a special micropore-forming capability, able to improve the mechanical properties of the construct while also promoting cell adhesion, growth and proliferation [175]. Liu et al. [173] prepared an ATP/GelMA composite hydrogel loaded with mouse BMSCs and murine umbilical vein ECs. In this study, the composite bioink exhibited a better printability and improved mechanical properties if compared to the neat GelMA bioink. Moreover, the cell-laden composite hydrogels could effectively enhance bone regeneration while also promoting angiogenesis after a 2-week implantation in vivo.

## In vivo advances in the 3D bioprinting of OC tissue

### Cellularized biomaterials

Three-dimensional bioprinting has recently gained significant attention for the repair of OC defects as it allows the manufacturing of scaffolds mimicking the high level of interfacial tissue organization and complexity to stimulate in vivo OC defect repair [26]. However, if compared to bone and cartilage, OC bioprinting has been explored to a minor extent, especially for in vivo applications (Table 3).

**Table 3:** Material composition, cell type and/or active molecule, animal model and main outcome of in vivo studies on 3D bioprinted OC constructs

**Material**		**3D (bio)printing technique**				**Cell type**		**Active molecule**		**Animal model**	**In vivo outcome**	**Ref.**
*Cartilage*	*Bone*	*Cartilage*			*Bone*	*Cartilage*	*Bone*	*Cartilage*	*Bone*			
Alg GelMA 2-Aminoethyl CSMA	Alg GelMA CSMA HAMA β-TCP	Microfluidic extrusion				Human MSCs ACs	Human MSCs	–		Orthotopic, rat (trochlear groove)	Co-culture enhanced CoL and aggrecan expression Reduction of hypertrophic differentiation of human MSCs	[182]^a^
Sodium Alg GeL	Sodium Alg GeL Hap	Extrusion				Rabbit cartilage–induced BMSCs	Rabbit osteogenic–induced BMSCs	–		Orthotopic, rabbit (trochlear groove)	Biomechanical integration after 3 months and 6 months Insufficient cartilage growth	[178]
GG Methyl cellulose Sodium Alg	GG Methyl cellulose Sodium Alg LMS	Extrusion				Rabbit CCs	Human placental MSCs	–		Orthotopic, rabbit (trochlear groove)	Absence of inflammation after 12 weeks LMS stimulated cellular regeneration ability of cartilage and bone	[181]
PNT	PNT β-TCP	Extrusion				–		TGF-β1	–	Orthotopic, rat (trochlear groove)	Cartilage and bone repair promoted by TGF-β1 and β-TCP, respectively	[184]
GelMA Cartilage dECM		SLA				–		Exosomes		Orthotopic, rabbit (trochlear groove)	Immune response control Antioxidative stress ability Increased bone formation	[179]
GelMA	PCL Hap	DLP	Melt extrusion			–		IL-4	–	Orthotopic, rabbit (trochlear groove)	High degradation of cartilage zone after 8 weeks Full bone growth after 16 weeks CC phenotype maintenance and enhanced cartilage repair by IL-4 release	[51]
GelMA SF	GelMA SFMA	Extrusion				–		PTH	–	Orthotopic, rabbit (trochlear groove)	No inflammation after 6 weeks and 12 weeks Uniform cartilage thickness Interlocking between bone trabeculae and cartilage	[180]
Cucurbit[6]uril CB[6] HA 1,6-Diaminohexane-conjugated HA PCL	CoL PCL	Dual extrusion (hydrogel extrusion + PCL melt extrusion)				Human turbinate-derived mesenchymal stromal cells		TGF-β	BMP2	Orthotopic, rabbit (trochlear groove)	No inflammation Scaffold integration at week 8	[187]
PCL HAMA	PCL β-TCP	Dual extrusion (hydrogel extrusion + PCL melt extrusion)				Human BMSCs Rat BMSCs	–	KGN DC	–	Orthotopic, rat (trochlear groove)	KGN increased BMSC-mediated cartilage regeneration Enhanced chondroprotective and inflammatory management of BMSCs after 12 weeks Improved subchondral bone regeneration by TCP and scaffold porosity	[52]
Cartilage dECM SF	Bone dECM SF PCL	Extrusion		Dual extrusion (hydrogel extrusion + PCL melt extrusion)		Rabbit BMSCs		TGF-β1	BMP2	Orthotopic, rabbit (trochlear groove)	Excellent scaffold integration OC regeneration	[186]
GelMA GelMA-DA Acrylate β-cyclodextrin	GelMA GelMA-DA Ac-β-CD	Extrusion				ADSCs		KTG	Melatonin	Orthotopic, rabbit (trochlear groove)	Synergic effect of hydrogel ADSCs and KGN on cartilage repair at 12 weeks High expression of CoL type I in subchondral bone	[183]

^a^The scaffolds developed in this work were designed for the regeneration of chondral defect, including calcified cartilage

Biphasic and triphasic cellularized scaffolds with distinct bone and cartilage phases were fabricated to mimic the different cartilage and subchondral bone compositions [178–180]. Indeed, two different cell types [181] or tissue-specific-induced BMSCs [178] were used in the different regions of the construct. For example, Yang and co-workers [178] printed a scaffold carrying cartilage-induced BMSCs within an Alg/GeL gel as the cartilage layer, and osteogenic-induced BMSCs within an Alg/GeL/Hap gel as the bone layer. In this way, it was possible to obtain a scaffold made of 14 layers of the bone region overlaid by 14 layers of the cartilage region and provided with 500 µm pore size through the entire thickness. At 6 months after orthotopic implantation in rabbits, the authors observed the almost complete repair of articular surface and subchondral bone. However, tissue ingrowth was incomplete, and the mechanical properties differed from normal cartilage tissue, suggesting the need of a longer implantation time for a final scaffold’s evaluation [178].

Table 3 highlights important trends with respect to cell type and source. In the case of the cartilage region, stem cells, from mesenchyme or bone marrow origin, and CCs were selected as first choice [178, 181, 182]. Moreover, one study also combined human articular CCs and human bone–derived MSCs [182]. This trend is in line with what reported in the section ‘In vivo advances in the 3D bioprinting of cartilage’ for cartilage. Similar cell densities, equal to 1 × 10^7^ cells/mL for CC/MSC co-culture [182] and to 3 × 10^7^ cells/mL for BMSCs, were used [178]. Minor cell density differences are normally observed between the cellularization of bone and cartilage regions.

The in vitro scaffold maturation before in vivo implantation was investigated in refs. [181] and [182], with different approaches. Qin and co-workers [181] cultured GG scaffolds loaded with rabbit CCs and rabbit BMSCs after printing, for a relatively short period, i.e. 3 days, to improve Li-Mg-Si (LMS) bioceramics-induced cellular proliferation. Conversely, Idaszek and co-workers [182] precultured the scaffolds for 3 months before in vivo implantation. Such a long period of scaffold maturation was performed to improve the scaffold mechanical strength [182]. In line with what discussed in the section ‘In vivo advances in the 3D bioprinting of cartilage’ also in the study of Idaszek et al. [182], co-culture of CCs with stem cells in a 1:3 ratio increased CC proliferation and deposition of cartilaginous ECM, while paracrine factors released by CCs enhanced human MSC chondrogenesis. Moreover, in the case of the gradient scaffold investigated in this study, articular CCs encapsulated in the hyaline cartilage layer reduced the hypertrophic differentiation of human MSCs encapsulated in the calcified zone.

As reported in Table 3, materials made of natural polymers or chemically modified natural polymers are widely used. Hydrogels made of e.g. Alg, GelMA, GeL and methyl cellulose were used as cell-laden bioinks to build the cartilaginous zone of OC scaffolds [178, 181, 182]. In fact, these materials may provide the ideal protein- and polysaccharide-rich hydrophilic microenvironment for cartilage and/or boost stem cells’ growth and biosynthesis. In contrast, hydrogels incorporating inorganic osteoinductive fillers were used in the case of the bone region of OC scaffolds [178, 181, 182].

The loading of inorganic osteoinductive fillers within the bone layers is employed to enhance both biomechanical and biological scaffold features. As also observed in the case of bone scaffolds (‘In vivo advances in the 3D bioprinting of bone’), nanometric or micrometric Hap and β-TCP particles have been used [178, 182]. The used concentration of β-TCP particles was 0.5 wt% [182], whereas in the case of Hap, a concentration of 4% of particles was used [178]. The use of inorganic µPs made of silicate bioceramics is advantageous for OC TE scaffolds as these biomaterials may release multiple ions with different bioactivities towards bone and cartilage tissue regeneration. Qin and co-workers [181] loaded 10 wt% of LMS particles within the bone region of a bilayer OC scaffolds made of GG/methyl cellulose/sodium Alg hydrogel. The Li and Si ions released from the bone region diffused to the cartilage region stimulating the in vitro maturation of encapsulated CCs. Furthermore, the released Si ions regulated the osteogenic gene expression of human placental MSCs encapsulated within the bone region. Meanwhile, Mg ions could promote the synthesis of cartilage matrix through integrins and stimulate the mineralization of calcified tissues [181]. It is worth noting that the choice of particle size and concentration into the bioconstruct depends not only on the bioactivation strategy, but also on processing and biocompatibility issues. In fact, the addition of an inorganic filler enhances bioink viscosity and, consequently, the shear stresses to encapsulated cells during printing [51]. Furthermore, filler release after polymeric matrix degradation may affect cell viability, especially in the case of nPs capable to be internalized by transplanted cells. These aspects require the assessment of cell viability in vitro before the in vivo implantation [182].

### Acellular biomaterials functionalized with active factors

Fabricating a microenvironment that mimics physiological settings by incorporating bioactive molecules, such as growth factors and drugs, into the scaffold, is a key aspect for OC TE [183]. Hydrogels made of synthetic and/or natural polymers, such as GelMA and SF, may combine bioactivity, printability properties and drug loading capability to stimulate proper tissue development after in vivo implantation [179, 180]. There are four reported works about the use of cell-free scaffolds bioactivated with biomolecules or exosomes (Table 3). Surprisingly, the analysis of the works evidenced that the biomolecules were loaded only within the cartilage region of the scaffolds, suggesting that drug delivery is a key issue especially for cartilage regeneration. This is probably because, as previously reported, the bioactivation of the bone region can be also achieved by the loading of bioactive inorganic fillers. Gao and co-workers [184] fabricated biphasic high-strength porous hydrogel scaffolds with TGF-β1 and β-TCP nPs in distinct layers, to stimulate cartilage and bone regeneration, respectively. A thermoresponsive supramolecular co-polymer hydrogel, named PNT hydrogel, featuring shear thinning property was synthesized by the co-polymerization of dual hydrogen-bonding monomers, *N*-acryloyl glycinamide (NAGA), and *N*-[tris(hydroxymethyl)methyl]acrylamide (THMMA). By the careful modulation of the NAGA/THMMA ratio, it was possible to tune the mechanical properties of the PNT hydrogel for the cartilage and bone compartment of OC scaffold [184]. Different concentrations of β-TCP nPs, in the 12.5 to 30 wt% range, were incorporated into the PNT hydrogel to form the bottom layers of 3D printed gradient scaffolds, thus enhancing integration with the host bone [184]. After 12 weeks of implantation in rats, the biohybrid scaffolds enabled the regeneration of a uniform and smooth layer of new cartilage well integrated with the subchondral bone, with no signs of bone overgrowth towards the cartilage region. Most importantly, the newly repaired cartilage tissue was composed of glycosaminoglycans and CoL type II, and the cell density in this layer was close to that of the native cartilage [184].

Drug loading was also employed to control the inflammatory microenvironment of the articular joint. Inflammatory conditions may occur as natural response to joint injuries, such as in the case of osteoarthritis, but are also the consequence of host response to surgical procedures and to implanted materials [51]. Cartilage breakdown fragments created by OC defects and medial meniscectomy surgeries, followed by the enzymatic or mechanical destruction of cartilage, can further trigger the release of hydrolytic enzymes (such as collagenase) from macrophages and synovial cells, resulting in the decrease of chondrogenesis [51, 52, 185]. The repair of OC defects in a progressive osteoarthritis inflammatory microenvironment remains unsatisfactory in the clinic [52]. To address this issue, Gong and co-workers [51] combined DLP and fused deposition modelling (FDM) techniques to fabricate a bilayer GelMA/PCL-Hap OC scaffold loaded with interleukin-4 (IL-4). IL-4 is a biomolecule involved in the immunomodulation of the macrophage phenotype towards an anti-inflammatory/regulatory M2 phenotype at the tissue-implant interface. Furthermore, a radially oriented porosity of GelMA hydrogel was optimized to enhance surrounding cell infiltration and migration into the hydrogel. IL-4 released from the cartilage layer reduced the negative effects of IL-1β and M1 macrophages on CCs in an in vitro inflammatory model. At 16 weeks post-surgery in a rabbit model, the IL-4-loaded scaffold stimulated the formation of hyaline cartilage–like tissue that covered the entire defect area, and full neo-bone tissue formation was observed in the PCL/HA region [51]. It is worth to note that this represents the only work that combined a drug-loaded hydrogel with a PCL network as reinforcing structure.

To overcome possible immune incompatibility and chromosomal aberrations of transplanted BMSCs, Chen and co-workers [179] loaded MSC-derived exosomes into cartilage ECM/GelMA bioprinted OC scaffolds. Exosomes are extracellular vesicles that possess analogous functions to the cells from which they are derived and were found to decrease the level of cartilage matrix degradation markers and to increase CoL and aggrecan expression [179]. When loaded into the scaffold, exosomes modulated the immune response by promoting M2 macrophage infiltration and reducing the level of malondialdehyde, an indicator of synovial fluid lipid peroxidation, in the synovial fluid at 12 weeks post-surgery. The antioxidative stress ability of exosomes effectively restored cartilage mitochondrial dysfunction and enhanced CC migration [179].

Terminal differentiation of CCs, such as hypertrophy and calcification, is responsible for the formation of fibrous cartilage and, ultimately, the significant reduction in the quality of repaired cartilage [180]. To solve this problem, Deng and co-workers fabricated a biphasic scaffold by mixing GelMA with either parathyroid hormone (PTH)-grafted SF or SFMA [180]. PTH was loaded into the cartilage zone to inhibit the hypertrophy of CCs and to maintain the phenotype of hyaline cartilage, while GM-SF-MA was used to enhance mechanical properties of the bone zone. After 12 weeks of implantation, morphological observation evidenced an intact and smooth cartilage surface, together with a uniform and continuous cartilage tissue. Furthermore, scaffold supported subchondral bone regeneration to a large extent and proper interlocking between new bone and surrounding native tissue [180].

### Synergies between cell therapy and drug delivery

**Fig. 5:**
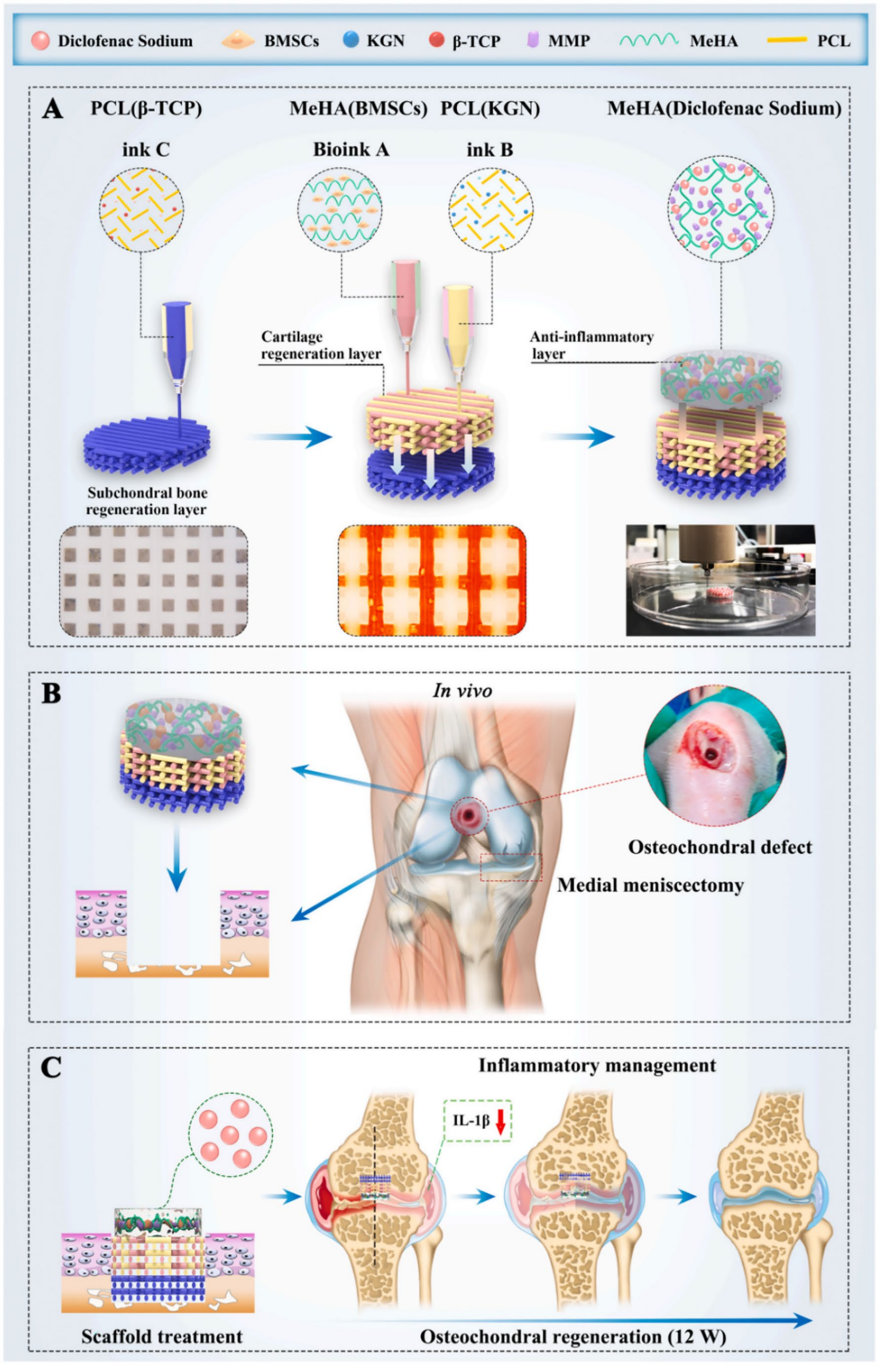
Flowchart of 3D bioprinted BMSC-laden biomimetic multiphasic scaffolds for OC defects in osteoarthritic joints. **A** The scaffold is composed of a PCL(β-TCP) porous structure to mimic the subchondral trabecular structure and a PCL(KGN) and BMSC-laden HAMA bioink with a multimaterial alternate printing pattern for cartilage regeneration; an MMP-HAMA(diclofenac (DC)) hydrogel layer coats the top layer to manage the inflammatory and pain effects of cartilage lesions with a disease-responsive pattern. Reproduced with permission from ref. [52] without any modification

Drug-loaded scaffolds were also designed to enhance the regenerative potential of encapsulated cells for OC tissue repair. Among the different biochemical cues, TGFs, BMP and kartogenin (KGN) were the most used for in vivo tissue regeneration [52, 183, 186, 187]. More in detail, the spatial organization of TGF-β and BMP2 within the cartilage and bone regions, respectively, was exploited to enhance OC regeneration in vivo [186]. Reported concentrations of TGF-β were in the range of 0.1–4 µm/mL, while in the case of BMP2, the concentrations were in the range of 4–5 µg/mL [186, 187]. KGN is a small molecule capable to upregulate chondrogenic gene expression and foster the selective differentiation of BMSCs towards CCs [52, 188]. As shown in Fig. 5, Liu and co-workers [52] proposed a cell-laden drug delivery scaffold capable to control early inflammation and to target cartilage catabolism, promoted by e.g. the secretion of matrix metalloproteinase (MMP) enzymes, after OC scaffold implantation.

The control of the inflammatory response was achieved by coating the top of the cartilage region with a 0.5-mm-thick DC-loaded hydrogel synthesized by using MMP-sensitive cleavable peptides. Such system was capable to regulate the release of the anti-inflammatory drug in response to the MMP levels (Fig. 5 and Table 3) [52]. To this aim, authors fabricated a three-layer scaffold as follows. The bone region was obtained by printing a PCL/β-TCP composite mimicking the subchondral trabecular structure, whereas the cartilage region consisted of a BMSC-laden HAMA hydrogel printed within the pores of KGN-loaded PCL. KGN was loaded into the cartilage region at 1 wt% to stimulate BMSC proliferation, migration and chondrogenic differentiation [52]. The results revealed chondroprotective and anti-inflammatory effects of the scaffold in vivo, even in the absence of the MMP-HAMA(DC) coating, finally suggesting that BMSCs themselves may inhibit inflammatory progression, either directly or indirectly [52]. Concomitantly, the scaffolds promoted subchondral bone regeneration in the bone region [52].

To better control the release kinetics of the active factors, drug delivery systems can be used instead of including the free active molecule in the scaffold. For example, Dai and co-workers [183] proposed a novel host–guest-modulated dynamic GelMA-based hydrogel. The hydrogel was functionalized with dopamine (GelMA-DA) and acrylate β-cyclodextrin to control the release of KGN and melatonin, an osteogenic factor, in the chondral and bone regions, respectively. The stiffness of the hydrogel was modulated by tuning the cross-linking density to match cartilage and bone regionality requirements. Most importantly, the different bioactivations of the chondral and bone regions enabled the use of a single cell type in the entire scaffold, namely ADSCs that have the potential to differentiate osteogenically or chondrogenically.

Importantly, most of the studies reported the use of a single cell type (MSCs) in both zones, highlighting that, when using active factors, these can stimulate the zonal bioactivation of undifferentiated cells and avoid the use of zone-specific cell types. For example, Shim and co-workers [187] employed human turbinate-derived mesenchymal stromal cells in both regions, where a zonal bioactivation was achieved by a spatial distribution of TGF-β and recombinant human BMP2. An advantage of utilizing biphasic spatially bioactivated scaffolds loaded with undifferentiated cells is that both phases of the construct can be maintained in the same culture conditions in vitro, as the approach relies on the cells within the two phases executing opposing programs in vivo [25, 187]. Following subcutaneous implantation, distinct tissues developed in vivo within the different regions of the biphasic implants. Scaffolds loaded with human turbinate-derived mesenchymal stromal cells decreased the inflammatory response and enhanced the integration with the surrounding cartilage tissue and the subchondral bone [187].

As already discussed in previous sections for cartilage and bone, also for the OC tissue, one of the most investigated strategies to improve the mechanical properties of hydrogel scaffolds involves the printing of a cell-laden hydrogel within a thermoplastic framework made of, e.g. PCL [25, 51, 52, 186, 187, 189]. Zhang and co-workers [186] extruded PCL to print the frame of the bone layer and filled the empty microchannels by printing a bone dECM/SF bioink. The cartilage region was made of a cartilage dECM/SF bioink printed on top of the bone region. To bioactivate the scaffolds, cartilage and bone hydrogels were loaded with TGF-β1 and BMP2, respectively, and both cellularized with BMSCs [186]. Although the scaffolds promoted OC regeneration in a rabbit knee joint model, a possible limitation of this approach is the excessive difference in the mechanical behaviour between the PCL-reinforced bone region and the PCL-free cartilage region. To overcome this limitation, the thermoplastic framework can be applied to the entire scaffold, while the chondrogenic and osteogenic environments can be designed by the optimization of the composition of the filling hydrogels [189]. This approach was used for instance by Shim and co-workers [187].

## Open questions and unmet challenges

**Fig. 6:**
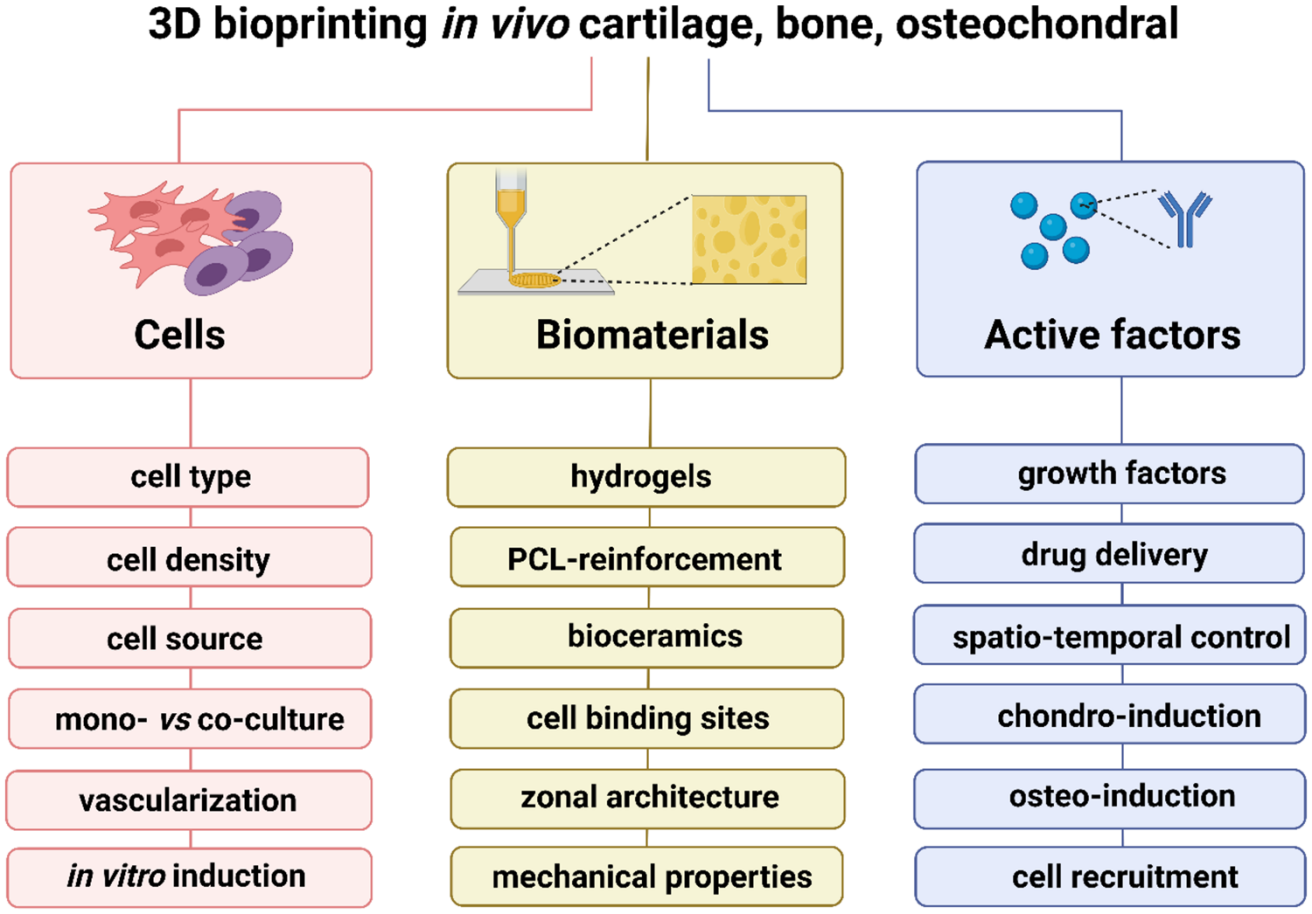
Crucial elements for the design of 3D bioprinted scaffolds intended for the in vivo regeneration of cartilage, bone and OC tissue

Based on the preceding discussion, it is evident that researchers in the field have successfully pinpointed a multitude of pivotal factors essential for the development of 3D bioprinted scaffolds, specifically designed for the in vivo regeneration of cartilage, bone and OC tissue. These elements are related to cells (e.g. cell type, use of co-culture), biomaterials (e.g. hydrogels, thermoplastic reinforcement) and bioactive molecules (e.g. use of drug delivery systems) (Fig. 6). However, a consensus regarding the optimal material combinations and procedures has yet to be achieved. In fact, despite the promising outcomes documented so far on the topic, a series of limitations, open research questions and unmet challenges persist. These are critically discussed in the following sections.

### Animal models

One of the points under discussion is the choice of the most appropriate animal model since an ideal preclinical in vivo model able to recapitulate the complexity of human cases does not exist [190]. For cartilage and bone applications, in vivo studies intended to evaluate 3D printed scaffolds loaded with cells and/or active factors employ either subcutaneous mouse/rat models or orthotopic models. The latter are mainly performed in small animals (mice or rats) for bone and in medium/large animals (rabbits, goats, sheep) for cartilage (Tables 1 and 2). In the case of OC tissue, only orthotopic (trochlear groove) defect models, mainly in rabbits, have been reported (Table 3). In general, a subcutaneous model is useful to test biocompatibility prior implantation in large animals and allows high throughput, lower cost and easier animal caging and handling compared to large animal models. Importantly, the availability of immunodeficient mice enables in vivo testing of human cells in a preclinical phase [68–72, 79, 83]. However, the diminished immune response can impact scaffold performance in terms of scaffold stability and the formation of new tissue in a manner that does not accurately represent scaffold performance in the presence of an active immune system. For instance, when comparing immunodeficient and immunocompetent mice, it was observed that new bone formation was lower during the first 4 weeks in the latter group. This difference was attributed to the influence of inflammatory cytokines and immune cells on the survival of transplanted cells [191]. Therefore, the use of both immunodeficient and immunocompetent mice may help to elucidate the role of the immunological response on new tissue formation. For bone applications, the subcutaneous model is also used to test vascularization prior the orthotopic model [160, 168, 176]. However, the lack of anatomic, biomechanical and biochemical similarities between the subcutaneous and orthotopic location renders this model insufficient for studying in vivo new tissue formation and mechanical performance of scaffolds [192]. Nevertheless, in the case of cartilage, several studies report new tissue formation in 3D bioprinted scaffolds implanted subcutaneously (Table 1). Unfortunately, these studies are not followed by further investigations of the same scaffolds in the orthotopic location of larger animals, which is mechanically more challenging and more representative of the final application. Conversely, in the case of bone, subcutaneous models are often followed by the evaluation of the same scaffold in an orthotopic model [104, 113, 119, 157, 160, 168, 176].

When using orthotopic models, the rabbit model is the most commonly used in cartilage, followed by the sheep and goat models (Table 1). This is because rabbits have sufficiently large cartilage surfaces where tissue defects can be created, while their housing and care remain relatively easy and cost-effective [193]. The main limitation here is that rabbit cartilage has a higher spontaneous regenerative ability and less mechanical challenge than those of humans [194]. For bone applications, the rat or mouse calvarium and the femur are the two most commonly employed orthotopic locations, followed by tibia, spine and mandibula (Table 2). The rat critical size calvarial defect is a widely used model due to its low cost, easiness in the defect generation and reproducibility [195]. Its high throughput allows the screening of a large number of biomaterial prototypes before large animal testing. Given that the calvarium is a non-load bearing location, this model is suitable for applications in the craniofacial bone regeneration, whereas to test scaffolds intended to bear high mechanical forces, long bone locations, such as the femur, are more appropriate [195]. Remarkably, apart from a few studies on medium-sized animals (rabbits), none of the studies on bone regeneration reported evaluation in large animal models.

In orthotopic models, the defect location is another important aspect. A strong relationship between healing capacity and defect location was reported in literature, as both surface geometry and load-bearing stresses can influence study outcomes [190]. In the case of OC scaffolds, there is a consensus on the in vivo implantation into defects created on the femoro-patellar groove of the knee joint instead of the femoral condyle. OC lesions of knee often occur in the condylar regions due to the higher articular loads compared to other regions. However, scaffold implantation into load-bearing articular regions is not only impaired by the biomechanical requirements, but it is also limited by ethical issues. In fact, in the case of the OC tissue, the articular joint should be immobilized until a strong scaffold-tissue bonding is achieved to reduce pain and avoid tissue damage and scaffold displacement into the joint cavity. Despite the recent advances, the design of bioconstructs capable to withstand shear and compression mechanical loads, from the surface down to the deeper bone region of the condylar site, is still challenging. In this context, if compared to condylar defects, those created into the trochlear groove region are technically easier to perform [190], while healing and scaffold integration are easier. This, on the one hand, explains why this defect location is often preferred; on the other hand, it triggers the question on whether materials tested in a femoro-patellar groove defect may display the same performance in a femoral condyle defect.

Our search revealed that there is no general consensus on what the most appropriate in vivo study duration for the different models is. The articles selected for this review report a quite broad time frame which ranges between 3 and 24 weeks for most of them, whereas only a few articles report a duration equal or longer than 6 months [71, 89, 90, 178]. Typically, short-term in vivo studies only score the quality of the regenerated cartilage using biochemical assays such as histology, immunohistochemistry and gene expression. However, the deposition of cartilage-like ECM is not sufficient to prove the formation of a functional repaired tissue. In contrast, one of the advantages of long-term in vivo studies is to test the improvement in mechanical properties of the newly formed tissue [71, 89, 90, 178], which is difficult to observe during short-term in vivo studies [76].

### In vitro scaffold maturation

An important open question is whether in vivo performance of 3D bioprinted scaffolds can benefit from a preliminary in vitro scaffold maturation, i.e. the in vitro preculture of cellularized scaffolds under chondrogenic/osteogenic conditions before in vivo scaffold implantation. This has been suggested as a promising strategy to boost cell differentiation and new tissue formation in cartilage, bone and OC scaffolds, and this, in turn, may shorten regeneration time in vivo [182, 196–198]. Among the studies analysed in this review, a few of them apply this strategy for bone [104, 106, 107, 120, 149, 176], cartilage [95] or the OC tissue [178, 181, 182] with a preculture period ranging between 24 h and 3 months. Importantly, the lack of comparative studies does not allow reaching a consensus on the effect of preculture on the in vivo scaffold performance. Future efforts in this direction will especially focus on what would be the ideal preculture time which will affect the degree of scaffold maturation. In fact, it has been reported that longer in vitro scaffold maturation, despite the higher histology score, can lead to lower scaffold integration with the surrounding tissue upon implantation [196].

### Bridging the gap between in vitro and in vivo models

While in vitro models are useful to study scaffold performance focusing on cell behaviour and ECM formation, they lack the complexity of the native tissue environment, and their translation to in vivo conditions is often challenging. On the other hand, in vivo models provide information about tissue integration, immune response and overall functionality, but they raise ethical concerns and are associated with high cost. Hence, ex vivo models are emerging as an intermediary approach that bridges the gap between in vitro and in vivo models. For examples, 3D bioprinted scaffolds have been integrated into tissue defects created on osteochondral explants that were further cultured in vitro under biomimetic conditions [199, 200]. The defect induced in such explants can be chondral or OC, making them suitable for cartilage, as well as for OC scaffolds [199, 200]. Ex vivo cultures are often carried out in bioreactors designed to maintain controlled conditions, such as temperature, humidity, nutrient supply and mechanical stimulation, such as shear stress or compression, to mimic the physiological loading experienced in vivo [199, 201, 202]. Since ex vivo models maintain tissue architecture and cellular interactions, they offer a more relevant representation of the in vivo environment compared to traditional in vitro models, providing additional information on scaffold integration with the surrounding tissue. Moreover, implementing ex vivo models before animal studies can potentially reduce the number of animals required for subsequent in vivo studies, which is in line with the principles of the 3Rs (replacement, reduction and refinement) for ethical animal research. However, it should be considered that ex vivo models only recreate certain aspects of the in vivo environment and, therefore, they still lack the full complexity of a living organism. Moreover, maintaining ex vivo cultures for a long period of time may be technically challenging.

### In vivo non-invasive monitoring

A general limitation of the histological and mechanical procedures used to monitor the performance of 3D bioprinted constructs in vivo is that they are invasive and destructive and require sample processing (e.g. paraffin fixation, staining) that may introduce artefacts [203]. In this context, there is an urgent need to develop efficient methods for the non-invasive in vivo monitoring of the structural changes (e.g. scaffold degradation, scaffold integration, tissue ingrowth) that 3D printed constructs undergo over time. A few studies analysed in this review report in vivo monitoring of implanted scaffolds. In the case of cartilage for example, magnetic resonance imaging (MRI) has been used to monitor the ingrowth of blood vessels within 3D bioprinted cartilage scaffolds [79]. In the case of bone, micro-computed tomography (micro-CT) is the most used technique, followed by MRI, fluorescence microscopy, bioluminescence imaging, angiography and manual assessment [65, 66, 118, 140, 156, 176]. When using 3D printed hydrogels for bone regeneration, a concrete challenge is to simultaneously visualize the degrading hydrogel, the new tissue formation and the bone surrounding the defect. Due to the different density levels and water contents of these elements, different techniques may be used for the proper visualization of each structure. In fact, for example, micro-CT is the gold standard to image hard tissues, but it is not ideal to monitor hydrogel degradation in vivo [108]. Conversely, T1-weighted MRI was found more appropriate than micro-CT to visualize hydrogel degradation, but gave insufficient visualization of the bony tissue [108]. Hence, the combination of T1-weighted MRI and ultra-short echo time (UTE) MRI has been suggested to simultaneously monitor hydrogel degradation and bony tissue, given that UTE MRI allows hard tissue visualization [108]. Moreover, when using hybrid scaffolds made for example by a PCL-based framework and a hydrogel, it is challenging to visualize and distinguish the two components that have both low X-ray absorption and low refractive index. In this context, a method has been proposed that is based on the synchrotron radiation inline phase-contrast imaging combined with CT. This is a promising technique since it allows the visualization of tissue constructs with low refractive indices and weak X-ray absorption, for which X-ray micro-CT usually provides poor-resolution imaging [86, 204].

### Graft-defect mismatch

From a technical point of view, most of the studies are based on the implantation of prefabricated scaffolds. Typically, the 3D bioprinted scaffold is fabricated using a bench-based 3D bioprinter and implanted in a defect which has been created to perfectly match the shape and the size of the construct. In a clinical setting, given the natural irregularity of tissue defects, this strategy would require a surgical correction of the defect prior the implantation to ensure a good graft-defect match. Alternatively, customized scaffolds may be fabricated using computer tomography and/or MRI scans of the defect. In this context, intraoperative bioprinting, i.e. bioprinting performed in a surgical setting directly on a live animal, is gaining a growing interest. Intraoperative bioprinting techniques can be classified into automated approaches that uses a 3D bioprinter or a robotic arm, and those based on portable bioprinting devices [205]. For example, Moncal et al. [148] used an in situ automated 3D bioprinting for the repair of calvarial defects in rats. In contrast, an example of a portable device is given by the study of Di Bella et al. [76], who designed a hand-held pen which is easily handled by the surgeon. Both the automated approaches and the portable device technique aim to repair and reconstruct tissue defects involving curved surfaces or even more intricate geometries that are difficult to be matched with conventional 3D printing techniques. However, their translation to the clinical setting is still considered challenging owing to safety and processing issues. For example, filament deposition and setting must be carried out onto the patient’s wounds with a constant temperature (37 °C) and blood, which may cause a collapse of the printed structure before cross-linking [206]. Moreover, in the case of the automated approaches, there remains the need to prove that the resolution limit of the imaging techniques that capture the defect topography will allow a perfect graft-defect match. Finally, in the case of portable devices, it is not possible to reproduce an accurate scaffold porosity and zonal distribution of cells which can be achieved with a 3D bioprinter.

### Zonality and scaffold architecture

Given the anisotropic structure of cartilage, bone and OC tissue, biomimicking this zone-dependent architecture is a promising strategy to achieve a functional regenerated tissue with biochemical and biomechanical properties similar to those of the native tissue [178–180]. In this context, 3D bioprinting is a unique tool for the fabrication of stratified or zonal scaffolds. The fabrication of hierarchically organized scaffold is especially important for OC scaffolds given the complexity of the OC interface. Based on the studies analysed in this review, zonality in OC scaffolds is induced by preparing biphasic constructs featuring a superficial cartilage layer on top of a subchondral bone layer. The two layers may have different biomaterial compositions, active factors and cell types (‘In vivo advances in the 3D bioprinting of OC tissue’). Despite the wide literature about the key role of porosity on cartilage and bone regeneration [73, 207], none of the works analysed in the OC part of this review focused on how porosity could be exploited to confer scaffold zonality. In fact, when extrusion printing was used, the same printing pattern was employed for both regions. This, in most of the works, was a 90° printing configuration with a strand size and spacing equal to 500 µm [51, 52, 180, 184, 187], and only in one study, it was a 120° hexagonal configuration [181]. In another work, the scaffold architecture was designed to have radially oriented pores to enhance cell and tissue ingrowth; however, no microarchitectural differences were designed in the cartilage and bone regions [179]. This consideration suggests the need of further evaluating the role of porosity and pore structure features on in vivo OC regeneration.

As for cartilage, in the last decade, significant advances have been made to introduce zonality by using at least three different strategies: (i) zone-dependent scaffold architecture/composition, (ii) zone-dependent active factor localization and (iii) zone-dependent cell type or cell subpopulations [43, 208, 209]. However, when we narrow our search to in vivo studies according to the selection criteria of this review, we observe that only few articles actually report a zonal approach [73, 91, 92]. In these works, zonality is induced by varying the pore size of the different layers (pore size is increased from top to bottom) [73], by distributing different growth factors in different zones [92] or by a combination of the two approaches [91] (Fig. 3). Surprisingly, none of the articles report a zonal approach based on different cell types or CC subpopulations. This trend highlights that significant efforts still should be made for the translation of zonal 3D bioprinting from the in vitro to the in vivo stage. There is a clear need to reach a consensus on what are the scaffold requirements in terms of zonal porosity and architecture, on what are the active factors (and their dose) able to induce zonality and on what are the standardized protocols for cell harvest, sorting and expansion to induce, maximize and maintain zonal cellular phenotypes [208].

The creation of zonally patterned scaffolds, including the hierarchical microchannel structure, is also critical for the growth of new bone tissue in vivo. As described in the section ‘In vivo advances in the 3D bioprinting of bone’, this was done with a major focus on defining vascularized and avascularised regions. For example, Piard et al. [119] co-extruded a combination of HUVECs and human MSCs to reproduce the pattern found in native osteons, whereas Sun et al. [157] combined the osteogenic potential of BMP4 with ECs and BMSCs in a 3D printed biomimetic scaffold with hierarchical central medullary canals, peripheral Haversian canals and transverse Volkmann canals. Moreover, Shen et al. [120] developed a dual-bioprinting method to simultaneously print BMSCs to promote osteogenesis and ECs to promote angiogenesis. Finally, Kérourédan et al. [66] attempted to reproduce the bone structure by printing different patterns of ECs into mouse calvarial bone defects prefilled with HUVECs and VEGF.

## Conclusions

Orthopaedic disorders are prevalent in modern societies, highlighting the need for improved surgical options to address AC and bone defects. One potential solution lies in the field of bioprinting, an additive manufacturing technique that offers the ability to fabricate 3D bioconstructs mimicking natural tissues. By carefully controlling the spatial arrangement of cells and bioactive cues, bioprinting holds promise for the in vivo regeneration of bone, cartilage and OC tissues. In this comprehensive review, we critically analysed the application of 3D bioprinting for in vivo tissue regeneration. Zonally arranged bioinks, which are functionalized with cells and/or bioactive factors, provide 3D microenvironments that closely resemble the zonality and bioactivity of native tissues. To achieve optimal outcomes, it is crucial to consider various factors related to cells (cell type, source and combination), as well as to biomaterials (e.g. hydrogels, thermoplastic reinforcement) and the spatio-temporal distribution of bioactive molecules. Additionally, specific drug delivery systems play a vital role in enhancing the therapeutic efficacy. While numerous promising options have been proposed, achieving a consensus on the ideal material combinations and procedures remains a challenge. Furthermore, several limitations, open research questions and unmet challenges persist. These include the limitations of animal models used for testing, refining pre- and post-implantation procedures, bridging the gap between in vitro and in vivo models, developing efficient non-invasive monitoring techniques, addressing graft-defect mismatches, and identifying the most suitable scaffold architecture and drug delivery systems. Addressing these issues will be crucial for advancing the field of 3D bioprinting in orthopaedic tissue regeneration.

## Data Availability

Data sharing is not applicable to this article as no datasets were generated during this study.

## Materials availability

Material sharing is not applicable, because the studies included in this review were collected based on an electronic literature search.

## Abbreviations

3D: Three-dimensional
AC: Articular cartilage
ADSCs: Adipose-derived stem cells
Alg: Alginate
AlgMA: Alginate methacrylate
ATP: Attapulgite
BMP: Bone morphogenetic protein
BMSCs: Bone marrow–derived mesenchymal stem cells
CAD: Computer-aided design
cBMC: chicken bone marrow cells
CCs: Chondrocytes
CLI: Clindamycin
CoL: Collagen
CPC: Calcium phosphate cement
CSMA: Chondroitin sulphate methacrylate
CTGF: Connective tissue growth factor
DC: Diclofenac
dECM: Decellularized extracellular matrix
DFO: Deferoxamine
DLP: Digital light processing
DoD: Drop‐on‐demand
DX: Doxycycline
ECM: Extracellular matrix
ECs: Endothelial cells
ESCs: Embryonic stem cells
Eth: Ethosomes
FDM: Fused deposition modelling
GDF: Growth differentiation factor
GeL: Gelatin
GelMa: Gelatin methacrylamide
GelMA-DA: Dopamine-modified gelatin methacrylamide
GET: Glycosaminoglycan-binding enhanced transduction
GG: Gellan gum
HA: Hyaluronic acid
HAMA: Methacrylate hyaluronic acid
Hap: Hydroxyapatite
HIF1-α: Hypoxia-inducible factor 1-α
HPCMA: Hydroxypropyl cellulose methacrylate
HUVECs: Human umbilical vein endothelial cells
ICT: Icaritin
IL-4: Interleukin-4
iPSCs: Induced pluripotent stem cells
KGN: Kartogenin
LAB: Laser-assisted bioprinting
LMS: Li-Mg-Si
Micro-CT: Micro-computed tomography
µPs: Microparticles
MMP: Matrix metalloproteinase
MRI: Magnetic resonance imaging
MSCs: Mesenchymal stem cells
NAGA: *N*-Acryloyl glycinamide
NC: Nanocellulose
NGF: Nerve grow factor
nPs: Nanoparticles
NS: Nanosilicate
OC: Osteochondral
PCL: Polycaprolactone
PDGF: Platelet-derived growth factor
PDLSCs: Periodontal ligament stem cells
PEG: Polyethylene glycol
PGA: Polyglycolic acid
PLA: Polylactic acid
PLGA: Poly(lactic-*co*-glycolic) acid
PTH: Parathyroid hormone
PVA: Polyvinyl alcohol
RGD: Arginylglycylaspartic acid
RSV: Resveratrol
RUNX2: Runt-related transcription factor 2
SF: Silk fibroin
SFMA: Methacrylated silk fibroin
SLA: Stereolithography
SV: Simvastatin
SVF: Stromal vascular fraction
TCP: Tricalcium phosphate
TDN: Tetrahedral DNA nanostructure
TE: Tissue engineering
TGF: Transforming growth factor
TGF: Transforming growth factor
THMMA: *N*-[Tris(hydroxymethyl)methyl]acrylamide
UTE: Ultra-short echo time
UV: Ultraviolet
VEGF: Vascular endothelial growth factor
